# A Longitudinal Early Patient Encounter Program Through a Lens of Relationship-Centered Care

**DOI:** 10.1007/s11606-025-09666-5

**Published:** 2025-08-12

**Authors:** Roy Claessen, Rik Engbers, Roland Laan, Saskia Middeldorp, Annelies van Ede, Petra van Gurp

**Affiliations:** 1 Department of Internal Medicine, Radboud University Medical Center, Nijmegen, The Netherlands; 2 Radboudumc Health Academy, Teaching Institute Radboud University Medical Center, Nijmegen, The Netherlands; 3 Department of Rheumatology, Radboud University Medical Center, Nijmegen, The Netherlands

**Keywords:** early patient contact, healthcare relationship(s), integrated care, relationship-centered care

## Abstract

**Background:**

Medical education must be aligned with an evolving healthcare environment, emphasizing person-centered, integrated care. Focusing on healthcare relationships in early patient contact (EPC) may help future healthcare professionals to better address patient needs.

**Objective:**

Evaluating a longitudinal early patient encounter program from a relationship-centered care (RCC) perspective.

**Design:**

In a longitudinal early patient encounter program, students engaged with an authentic healthcare network through repeated encounters with a patient, the inner circle, and healthcare professionals. The program aimed to foster students’ understanding of healthcare, and to promote a holistic approach of healthcare by incorporating diverse perspectives within the healthcare network, including their own perspective. We conducted a template analysis of a representative sample of students’ learning reports, guided by relationship-centered care theory.

**Participants:**

First- and second-year undergraduate medical students at the Radboud University Medical Center, the Netherlands.

**Main Measures:**

Our main goal was to explore students’ learning about healthcare relationships using the RCC framework. We characterized their learning in terms of knowledge, skills, attitude, and competences.

**Key Results:**

Student learning was identified in all four dimensions of RCC: unique personhood, empathy, reciprocal learning, and benefit to patient care. They learned from both formal and informal participants within the healthcare network. Students gained knowledge, skills, attitude, and competences regarding healthcare relationships. Authentical reflection fostered students’ awareness of both professional and personal identity formation.

**Conclusions:**

Applying a RCC perspective can reveal valuable learning outcomes in a longitudinal early patient encounter program. This program enabled students to engage in and learn about holistic, empathic, reciprocal, and meaningful relationships. We hypothesize that this will enable them to better respond to current healthcare challenges like providing person-centered, integrated care.

**Supplementary Information:**

The online version contains supplementary material available at 10.1007/s11606-025-09666-5.

## INTRODUCTION

In 2015, the World Health Organization (WHO) introduced a framework on integrated, more person-centered care to adapt to a changing healthcare environment^1^. Modern healthcare systems are becoming increasingly complex, due to factors such as an aging population, a growing burden of multimorbidity, and the involvement of multiple providers across both hospital and non-hospital settings^2^. In addition to this complexity, artificial intelligence and resource shortages impose a risk of losing the human aspects of healthcare, impacting both patients’ needs and desires, as well as the professional identity of healthcare professionals.

Person-centered care seeks to address the human dimensions of healthcare and has been acknowledged for its beneficial effects on patient outcomes, satisfaction, and overall healthcare efficiency^3^. While the WHO framework predominantly emphasizes the isolated perspective of patients, families, and communities, the framework of relationship-centered care (RCC) offers a more dynamic lens. By focusing on the relationships among all individuals involved in healthcare, RCC encompasses both individual and interpersonal dimensions inherent in the complex multi-person healthcare network that characterizes modern healthcare systems. RCC focuses on the quality of relationships, including those between patients, relatives, caregivers, and healthcare professionals. RCC is based on four key dimensions: (1) recognition of personhood; (2) empathy as an integral component of the relationship; (3) reciprocity, i.e., both parties contribute to and are influenced by the relationship, leading to mutual benefits such as improved patient outcomes and enhanced well-being of healthcare professionals^4^; and (4) benefit to patient care^5^. Pursuing a more relational approach is expected to positively impact both patient experience and healthcare relationships^6,7^.

Within the context of a complex healthcare system, characterized by evolving needs, desires, and perspectives, medical education serves as a pivotal element in effectively preparing future healthcare professionals^8,9^. Various medical curricula have aimed to address the importance of interpersonal relationships for healthcare, primarily by fostering student-patient encounters, actively involving patients in the educational process, and reorienting the curriculum towards a more person-centered approach^10,11^. However, evidence on what and how students learn from these experiences remains scarce^12,13^. Common learning outcome measures such as student grades, student satisfaction, patient satisfaction, and value-based outcomes often reflect a one-sided perspective. To fully capture the complexity of multi-sided healthcare relationships, the RCC framework offers a more holistic approach^14–17^. While the overall educational benefits of early patient contact (EPC) in introducing the patient perspective are well recognized, EPC may also be a powerful tool for teaching students how to build meaningful relationships within healthcare environments.

We therefore introduced this RCC framework to study student learning during a longitudinal early patient encounter program. In this program, all first- and second-year undergraduate medical students at the Radboud University, Nijmegen, the Netherlands, step into a patient’s life, engage in repeated encounters, and interact with formal and informal participants within the healthcare network. A description of the program is provided in Box 1. The purpose of this study was to gain a better understanding of what and how students learn about establishing and maintaining relationships in an authentic healthcare context. To achieve this, we evaluated the learning outcomes of this program using the dimensions of RCC and explored how this learning contributed to students’ competence development.

Box 1 Outline of the longitudinal early patient encounter program.

**Table:** 

BACKGROUND Preparing medical students for their future role as healthcare professional requires attention to adaptive capacity, dealing with uncertainties, collaboration, and reflection, in addition to traditional knowledge transfer and skill training. Interaction with patients early in their education, as a first- or second-year student with minimal clinical experience, plays a crucial role in shaping their professional identity. It fosters a sense of responsibility and a deeper understanding of their future role in healthcare, incorporating the patient perspective. Early exposure stimulates students’ exploration of attitudes and values, aligning them with the patient needs and the professional and ethical standards of the medical profession	
VISION Patients serve as partners in medical education, helping to highlight the patient perspective Early exposure to authentic healthcare practice inspires and motivates students When students engage with patients early in their training, they experience reduced anxiety and increased confidence in their ability to interact with patients later in their education. Gradual exposure allows them to build their competence and comfort in clinical settings over time	
LEARNING GOALS The student… …establishes and maintains contact with a patient to explore their perspective …can identify, appreciate, and discuss the impact of health issues with the patient, connecting these experiences to medical practice and research …uses learning experiences to shape and explore their professional identity …seeks feedback from patient and persons in the healthcare network about their attitude and behavior as an aspiring professional …reflects on their own professional perspective and adjusts expectations/goals if needed	
LEARNING ACTIVITIES Meeting a patient at least six times in their authentic environment and healthcare setting provides the opportunity to: – engage in dialogue to explore the patient’s perspective on health, disease, healthcare, and their personal needs and preferences as well as those of others in the healthcare network – practice communication skills – observe authentic healthcare practice and interactions within the healthcare network A reflection assignment invites the student to reflect on the years’ experience and their professional development Possibility to continue with the same patient in next years’ program CONNECTION TO THE MEDICAL CURRICULUM Students were not introduced into the theoretical framework of relationship-centered care as such before attending this program. However, this learning program runs alongside other ongoing learning programs of the undergraduate curriculum, in which communication and empathy are emphasized. All learning activities are designed in line with the ‘Medical Training Framework, 2020′^18^. For a description of the medical curriculum in at the Radboud University Medical Center, more details can be found in the supplementary materials	

## METHODS

### Study Context

In this qualitative study, we explored a longitudinal early patient encounter program, in the first 2 years of the undergraduate medical curriculum of the Radboud University Medical Center, Nijmegen, the Netherlands. Patient encounters were self-organized by the students without faculty interference and took place within a patient’s authentic environment. Students contacted a patient by exploring their own personal networks, such as family members, friends, or acquaintances, or by obtaining approval to access a peer’s personal network. Patients participated voluntarily and did not receive financial compensation. At the end of the program students reflected on their experiences and learning outcomes through a mandatory written report. Open-ended questions were used to guide students, fostering authentic reflection.

### Data Collection

We included written reports from different cohorts. We separated first- and second-year students of four consecutive academic years (2018 until 2022). In each academic year, a maximum number of 330 students attended the program. We distinguished first- and second-year students since learning experiences might differ. We analyzed data from before, during, and after the Covid-19 pandemic, recognizing that changes in healthcare and disease concepts, along with restrictions on physical interactions, could have impacted how the learning program and healthcare in general were perceived. Only the cohort 2019–2020, year 2 was excluded, as the program and assessment for this group was significantly adapted due to pandemic circumstances. Given the data volume, we randomly selected an even sample from each remaining cohort. By performing this purposive sampling, we ensured a representative composition and inclusion of all relevant cohorts.

**Fig. 1:**
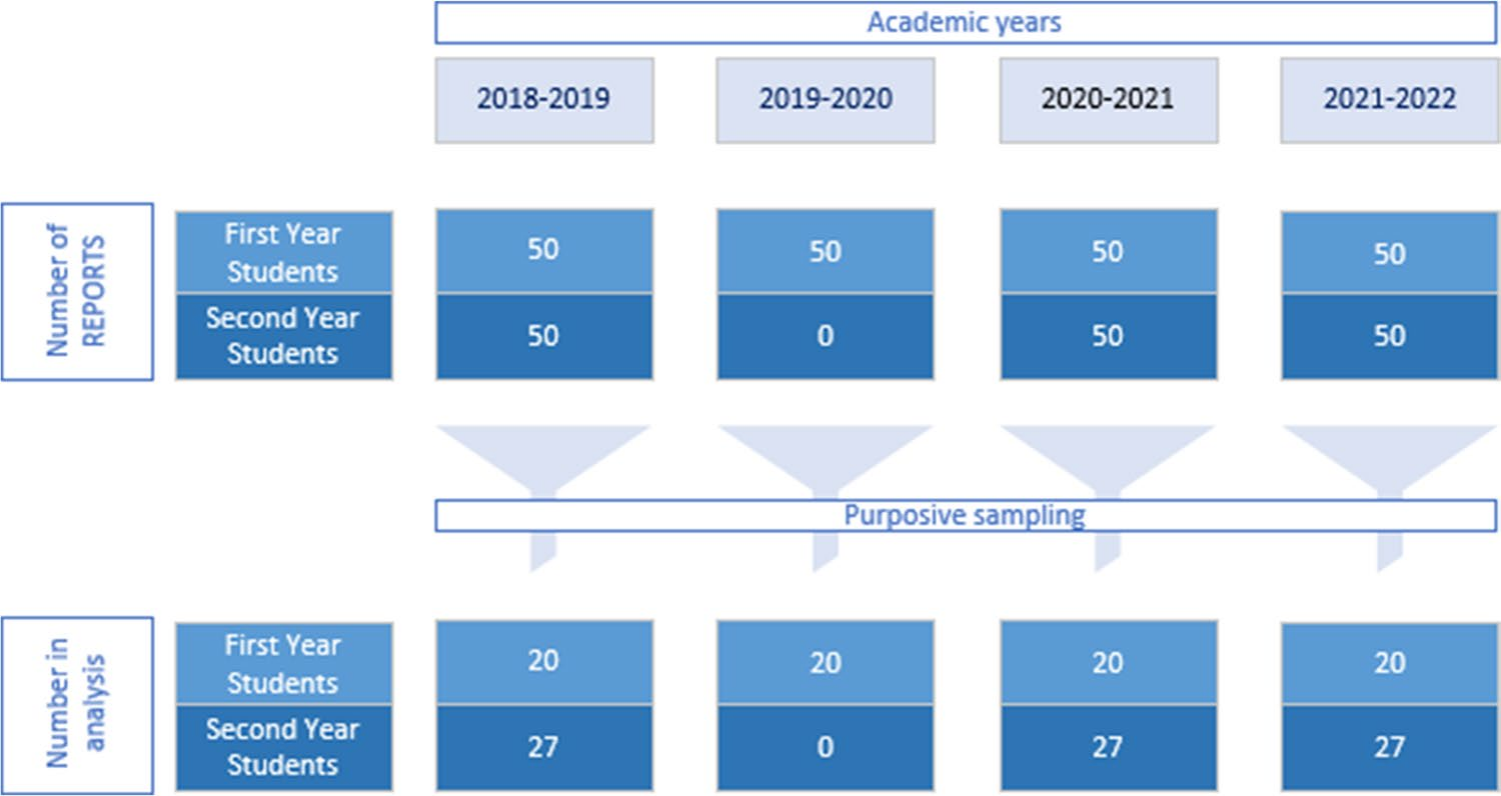
Composition of datafile.

Data were anonymized by the principal researcher (RC), to prevent identification of patients and students. To ensure feasibility with a large dataset, we created a first datafile of 161 randomly selected reports, aiming for the best possible representative sample. Figure 1 shows the composition of our datafile. Each individual report was indicated with a number, so references could be made.

### Data Analysis

Qualitative data was analyzed using Atlas.ti (version 24.0.0). Two researchers (RC, RE) conducted open thematic coding on 20 reports to familiarize themselves with the data and asses the usability of a RCC lens. Then we used a template analysis method. This method, described by King, thematically organizes and analyses qualitative data^19^. Based on key themes identified by the researcher as relevant to the research question, a coding template is developed. In template analysis, these themes or codes are then identified in the data, and the template can be reorganized to meaningfully represent the relationship between different themes. We used the theoretical framework of RCC as illustrated by Beach et al.^16^, as a starting point for our template. All known elements of RCC were transformed into separate codes to compose an initial template. Four researchers (RC, RE, AE, PG) independently used this template to analyze small sets of data. Results were cross-checked and discussed within the research group. Our initial template was grounded from theory, predominantly from a clinicians’ perspective. However, in the students’ program, due to its aims and design, we identified additional relevant themes related to RCC. Therefore, the template was adapted repeatedly in an iterative analytic process. When no new codes emerged, after consensus among the researchers, a final template was considered applicable to the data. Template evolvement can be found in the supplementary materials. The main researcher (RC) continued iterative analysis of the reports, cross-checking the results within the research group. After analyzing 120 of the 161 reports, consensus about data saturation was reached within the research group. To illustrate our results, we provide quotes, translated from Dutch to English.

We will present the results in two distinct ways. First, we identified students’ described learning experiences in 25 different themes related to the four main dimensions of RCC: unique personhood, empathy, reciprocal character, and benefit to care. To illustrate the richness of the learning outcomes, we will present a narrative per RCC dimension, incorporating quotes that refer to the different RCC themes. Second, we categorized learning experiences as belonging to different domains: knowledge, skills, attitudes, and competence. *Knowledge* refers to learning outcomes described as learning facts and awareness. *Skills* refer to learning outcomes described as learned how to do or how to handle (abilities). *Attitudes* refer to learning outcomes described as development of a new or different viewpoint or belief (values). *Competence* refers to the capability to fit knowledge, skills, and attitude in a specific context that leads to behavior that meets the specific demands. We will also indicate how this learning relates to the different RCC themes, and from whom within the healthcare network this was learned. The results of this analysis are summarized in a table.

Students’ described learning experiences were only allocated to a specific theme, dimension, or domain with consensus in the research group. In case of incidental doubt or potential assumptions, we refrained from allocating it, as this is an exploratory study aimed to qualify and not quantify learning outcomes.

## RESULTS

The distribution of the included reports was as follows: 48% first-year students, 52% second-year students, 64% female students, 36% male students. Additionally, 25% of students selected a patient they already knew, with 11% choosing a family member. One in five second-year students continued meeting the same patient as the previous year. The reports revealed healthcare situations involving various healthcare professionals, including medical doctors, specialized nurses, nurses, and paramedics. Almost all students described learning experiences related to unique personhood, empathy, and the benefit for healthcare. Fewer reports mentioned reciprocal character. The number of distinct learning experiences related to RCC ranged from 5 to 16 per written report.

### Narrative Per RCC Dimension

The narrative is based on quotes, extracted from various student reports, marked by ‘SR’ followed by the corresponding report number in our database. The number in brackets within the quotes refers to the RCC theme listed in the table.

#### Learning About Unique Personhood

Students gained knowledge regarding the unique personhood of patients, and its importance in establishing meaningful relationships. This knowledge contributed to students’ attitude concerning healthcare relationships (SR12):

*I learned a lot about how to view the patient as a person (1) and not just focus on the complaint or disease (4). There are often many more factors that affect a patient’s life and sometimes the complaint as well (24, 25). These are important things to consider as a physician when looking at treatment options (9, 12).*

Students developed an attitude to value the mutual contribution of each participant in a relationship, including the role of their own personality (SR48):

*You can only achieve an open relationship when both patient and doctor (honestly) tell their story and express their willingness to do so. That is not the responsibility of just the patient (2, 3).*

Students learned skills that were helpful in learning competences regarding unique personhood (SR8) and (SR35):

*I have also been open towards her from the beginning. By telling things about myself, I also wanted to make it easier for her to tell me about her illness (2,3,7)*

*By just talking and having conversations, showing that you are listening and continuing asking questions (4,5), you build a bond, and so you get the opportunity to get a complete picture of someone’s disease course and difficulties. You can empathize better that way (6), and so the relationship also shifts a bit from patient and doctor/student to somewhat more of a one-to-one conversation (3). *

#### Learning About Empathy

Students were exposed to emotional situations, calling on their empathic capabilities and creating awareness about the power of empathy in healthcare, both in their own relation with the patient and in observed healthcare situations (SR78) and (SR73):

*This really touched me because I had never thought about how hard it must have been for the patient and I felt deeply sorry for her (6). I also felt honored that she wanted to share this with me because it was very personal after all (5).*

*For myself I also experienced the cardiologist as an empathetic doctor who listened carefully to my patient (6).*

Some students described a struggle regarding emotional situations, their empathic attitude and behavior (SR75):

*At the phrase ‘there is a lot of fear I also got tears in my eyes immediately, but I did everything I could to suppress this. I felt as if in my role at that moment I was not allowed to show that this also affected me a lot, because I am trained as a doctor and doctors are not supposed to do this (20, 23). Empathy is good, but you also should not bring home too much misery as a doctor. Later, I also wrote a reflection about this (22).*

Where other students used earlier learning experiences in future interactions with patients (SR51):

*Last year I left the patient’s emotions out a bit, even though they are also especially important. I noticed that I still find it difficult or perhaps a little uncomfortable to ask about them. So, this year I have tried to work on this more. During the first conversation at the patient’s home this year, I started doing this. I did this by being honest with the patient and saying,"I would also like to ask some questions about the emotions surrounding the illness and I notice that I find this a bit difficult, would you mind if I ask about this? (5) The patient responded very positively to this, and we then had an open conversation. I noticed that I really liked this, and it helped me a lot.*

#### Reciprocal Learning in Healthcare Relationships

In the context of RCC, reciprocal learning refers to a situation where two individuals influence one another, resulting in mutual learning and growth. Students noticed that learning occurred in collaboration with the patient, but at the same time could be affected by the patient (SR12).

*The interviews went a lot easier this year. This also partly depends on the fact that my patient in year 1 was a bit less talkative (1,2,3’).*

Along their journey, students experienced what qualification was needed to effectively learn in collaboration with their patients (SR12):

*I found it difficult to start conversations last year and often did not know what to talk about (4). As a result, conversations were often short and to the point, and in retrospect I think I could have gotten more out of them (22). I have learned that through good preparation, conversations go easier because you already know what to talk about. Asking open-ended questions and then asking further also helps tremendously (7).*

The reciprocal character was further illustrated by patients and students sharing experiences (SR26) (SR73):

*During the first phone call with the doctor I (student) noticed that the patient had difficulties asking questions (22). I discussed this with the patient afterwards. The patient got the feeling that the doctor was in a hurry because the doctor called later than planned. The patient did not feel invited to ask questions (7,8,14). I also got this feeling while attending the phone call.*

*We reflected together on medical encounters (22), where she knew exactly which aspects she liked or not. She enabled me to ask questions about it. She also occasionally asked my perspective and responded with her perspective (22).*

We perceived that the student-patient encounter could be beneficial for patient’s knowledge or beliefs (SR71):

*I (student) found out that it is important to check whether the patient understands the information. My patient suffered from anemia and said he needed to eat a lot of potassium enriched vegetables. This did not seem logical to me, and I checked it with a doctor.*

And in behavior of patients in their relationship with healthcare professionals in a consultation (SR120):

*I tried to help my patient speak up more last year (11,12,14). She did not agree with a doctor’s decision then but did not dare to express it. When we stood outside after the appointment and talked about it, she told me she did not agree with the doctor. I then encouraged her not to be afraid of the doctor and just say what she wants to say, which she did in the next appointment.*

#### Learning About Benefits to Patient Care

Our data yielded hints about students experiencing that healthcare relations affected feelings, actions, and behavior on the patient’s behalf. We perceived that students developed an attitude about the importance of a good relationship with a healthcare professional or healthcare in general, potentially affecting a patient’s behavior, for the benefit of the patient (SR17).

*Still, she (the patient) is not very satisfied with care (13). The pediatrician my patient often visits makes her dislike the appointments. She does not get along very well with the doctor and she told me she experiences the doctor as very impersonal (7).*

*She also did not feel like talking much during consultations with the doctor or asking questions (12,14). Since she does not use ICT, social media or apps regarding her condition, unanswered questions persist. She told me these are often personal questions and because her mother always accompanies her to consultations (24), she is afraid to ask the doctor (14).*

### Domains of Learning Experiences

The table provides a summary of students’ perceived learning across 25 different RCC themes. In addition to the patient, students also interacted with individuals within the patient’s inner circle and healthcare network, including relatives, informal caregivers, healthcare professionals, and the healthcare community (including healthcare system facilities, insurance, policy makers). Accordingly, the table also indicates from whom students explicitly reported learning, marked by specific symbols. These symbols reflect the presence of these actors in the students’ written reports, without aiming to quantify how many students reported each source. We illustrate the table as follows: considering RCC theme 1, ‘Every patient has a unique personhood…perspective/culture/personality': In their written assignments, students reported to have gained knowledge, skills, attitude, and competence and to have learned about this theme by meeting the patient. Students also reported to have gained knowledge about this theme by meeting patient, relative, healthcare professional, and healthcare community.

**Table:**
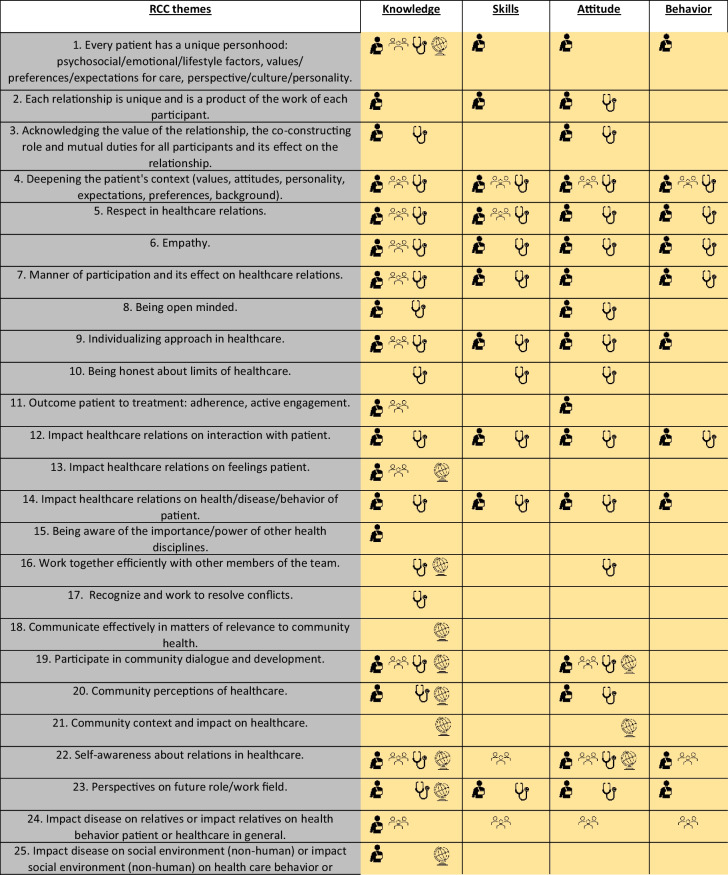


Students’ described learning experiences per RCC theme (left column) and their classification as pertaining to knowledge, skills, attitude, or competence, and from which participant in healthcare (corresponding symbol) this was learned:  : patient;  : relative;  : healthcare professional;  : healthcare community

## DISCUSSION

We used RCC perspectives to analyze the written reflection of students who participated in a program that asked them to engage in an authentic healthcare setting, in order to uncover what they learned about healthcare relationships and from whom they gained these insights. While EPC is already recognized for its potential to connect students with the healthcare community and deepen their understanding of healthcare, this program went a step further by immersing students in the actuality of healthcare setting^10,14,20,21^. Here, they engaged with others, were observed in practice, and gained insights into all four dimensions of relationship-centered care (RCC). We were able to describe students’ perceived learning in terms of knowledge, attitude, skills, and competence to engage in meaningful, empathic, reciprocal relationships in healthcare. Students recognized and appreciated the potential of healthcare relationships as key element of person-centered, integrated care.

Through this program, students became increasingly aware of what truly matters in healthcare relationships, not only for patients, but also in relation to their own emerging professional identities. In a context of limited experience and feelings of uncertainty, students were immersed in a complex environment of real-world encounters and dialogues. This program enabled students to meaningfully engage with and learn about healthcare relationships. Through authentic reflection, students gained valuable insight into both their personal and professional learning needs and areas for growth.

By fostering awareness and reflexivity about the complexity of healthcare relationships, we feel this program can help prepare students for the ongoing challenges in healthcare. Shifts towards a more active, informed, and decision-making role for patients, along with the increasing importance of multiprofessional team effort in healthcare, demand stronger collaboration and a more personalized, integrated approach to care. These challenges underscore the need for adaptive professionals capable of navigating complex healthcare environments^22^. The incorporation of collaboration with patients, engagement with a diverse range of healthcare professionals, and the development of adaptive expertise within medical curricula to meet the needs of the future healthcare environment has already been addressed^23,24^. This program aligns with literature that advocates for educational fundamentals that promote adaptive expertise development, including a focus on understanding rather than performance, active learning through struggle and discovery, and meaningful variation within an authentic healthcare environment^25^. Experiencing the complexity of real-world healthcare in a self-directed learning climate with minimal faculty guidance enabled students to encounter in unexpected situations with patients. This fostered our students to tap into and further develop adaptive skills and promoted a reflective attitude.

Given the widespread use and appreciation of EPC, we think our study can inspire other medical faculties in their attempt to adapt to actuality and simultaneously innovate in teaching methodology, to face the challenge of making a medical curriculum future proof. This student-led and self-planned learning program, with its novelty and uncertain expectations, provided students with rich learning experiences. The fact that this program was not primarily designed from a RCC perspective, for students not introduced to its principles, underlines the potential and feasibility of RCC in medical curricula, even in education that is not specially designed for it. As far as we know, an explicit RCC approach as used in this study to explore learning outcomes does not seem common in the design or formulation of learning goals of EPC in medical curricula. However, adopting a RCC perspective is feasible and applicable and it can provide valuable insights into how medical education has the potential to address the core element of relationships in healthcare.

### Strengths and Limitations

Our study’s strength lies in its use of a representative sample from a large student cohort in an ongoing learning program, grounded from a relevant theoretical framework with practical implications for healthcare and education. The findings may guide medical faculties in designing curricula that emphasize the impact of healthcare relationships.

Limitations include potential bias from socially desirable responses, a focus on students’ perspectives with limited patient or peer feedback, the influence of writing instructions, and students’ writing abilities. Due to the decision to analyze student self-reports, it was not possible to verify students’ self-reported learning outcomes. Although reciprocal learning was suggested, patient perspectives were not directly captured.

### Future Directions

Future research is needed to explore other teaching methodologies to incorporate the ideas of RCC in medical curricula. The “how” of learning RCC competencies, considering the interactions among different healthcare participants is still to be studied. Future research should also explore how RCC-focused learning activities can be assessed through formative assessment-for-learning approaches. We plan interviews with student-patient couples to capture dynamic interactions and learning outcomes.

In summary, viewing a longitudinal early patient contact program form a relationship-centered care perspective revealed valuable insight in students’ learning about healthcare relationships in a complex, holistic healthcare network. To align medical education with actual changes in healthcare, initiatives like ours introduce a valuable concept to evaluate or design medical curricula to prepare medical students for their professional future.

## Supplementary Information

Below is the link to the electronic supplementary material.

ESM 1 (DOCX 53.4 KB)

ESM 2 (DOCX 79.6 KB)
