# Supplementary material for: A Longitudinal Early Patient Encounter Program Through a Lens of Relationship-Centered Care
Source: J Gen Intern Med. 2025 Aug 12;40(16):3862–9. doi: 10.1007/s11606-025-09666-5 (PMC12686266; doi:10.1007/s11606-025-09666-5)
Supplement: Supplementary file 1 — (DOCX 53.4 KB) [file 11606_2025_9666_MOESM1_ESM.docx]

Appendix 1: Template development

Changes per version are described in grey text in general and marked in red in the schematic overview.

**Template version 1**

In version 1, the themes as indicated by Beach et al were used to analyze our data from a RCC perspective. We used this template to analyze the first twenty reports.

1. Clinician-patient, domain knowledge
   1. Each patient is a unique individual
   2. Psychosocial, emotional, and lifestyle issues are integral to medical care
   3. Patients differ in their values, preferences, and expectations for care
   4. Patients’ perspective, culture, and personality are relevant to the process of care
      1. "Each relationship is unique and is a product of the work of each participant"
   5. The manner in which a clinician participates in an encounter fundamentally affects the course, direction, and outcomes of care both episodically and longitudinally
2. Clinician-patient, domain approach, philosophy and attitudes
   1. "Value partnership with patients
   2. View patients as experts
   3. Acknowledge that patients deserve respect
   4. View the provider-patient relationship as a therapeutic vehicle
   5. Value the achievement of mutual respect and unconditional positive regard
   6. Acknowledge that affective engagement, rather than affective neutrality or detached concern, can further the therapeutic bond and its efficacy
   7. Acknowledge that clinicians and patients are both active human participants (not just role occupants) who co-construct their relationships
   8. Acknowledge that relationships are reciprocal and involve mutual tasks, duties an responsibilities
3. Clinician-patient, domain behaviors
   1. Show respect to patients
   2. Find out about patient’s values, expectations, preferences, and background
   3. Tailor approach to the patient based on knowledge of patient
   4. Help patient get story across, listen well (nonjudgmentally)
   5. Respond to patient’s emotions, show empathy
   6. Seek common ground as a point of departure for formulating therapeutic plans
   7. Attend to/monitor one’s own behavior as an influence on the other(s)
   8. Be aware of and acknowledge own feelings and biases (emotional self-awareness)
   9. Acknowledge the importance of relationships to the therapeutic process and outcome for both partners
   10. Acknowledge need to take both participants’ values, attitudes, and personality into account
   11. Acknowledge areas of agreement and disagreement on values, expectations, etc.
   12. Monitor the state of the relationship
   13. Acknowledge the importance of the relationship to one’s own well-being
4. Clinician-patient, domain outcomes
   1. Patient feels honored, respected, attended to, etc
   2. Patient likes and is satisfied with provider
   3. Patient has lower anxiety
   4. Patient has trust in provider
   5. Patient adheres to treatment
   6. Patient remembers information, advice
   7. Patient is more actively engaged
   8. Mutual attunement and harmony
   9. Informed decision making
   10. Added depth and vitality to interactions
   11. Clinician becomes a source of social and emotional support for the patient
   12. Patient becomes a source of professional reward/gratification for the clinician
   13. Protection against professional burnout
   14. Greater agreement on treatment plans
5. Clinician-clinician, domain knowledge
   1. Power inequities across health disciplines
   2. Power of understanding the other’s perspective
   3. Healing approaches of various health disciplines
   4. Team-building dynamics and approaches to shared leadership
6. Clinician-clinician relationship, domain approach, philosophy and attitudes
   1. Affirm importance of self awareness
   2. Value diversity and interdisciplinarity
   3. Appreciate importance of shared mission
   4. Is open to others’ ideas
   5. Affirm importance of, mutual respect, and trust
   6. Believe in importance of sustaining capacity for recognition, reconciliation, and prevention of error
7. Clinician-clinician relationship, domain behaviors
   1. Reflect on self and personal/ professional needs
   2. Continually learn from personal experience and that of others
   3. Learn cooperatively
   4. Derive personal meaning from the work of others
   5. Communicate effectively to other members of the team
   6. Listen actively to understand and engage other members of the team
   7. Work collaboratively, share responsibility
   8. Recognize and work to resolve conflicts
   9. Provide space in meetings for new thoughts, ideas
   10. Employ appreciative inquiry to imagine improvements
   11. Continuously examine whether the organizational values are reflected in day-to-day work
8. Clinician-clinician relationship, domain outcomes
   1. Productive resolution of disagreements
   2. Minimal staff turnover Improved ease of staff recruitment
   3. Colleagues reach personal and professional goals regularly
   4. Team members report being treated fairly and respectfully
   5. Enhanced capacity for working across a broad array of challenges
   6. Enhanced patient safety and quality of care
9. Clinician-community relationship, domain knowledge
   1. Diverse constructs/models of community
   2. Community perceptions of healthcare (including myths and misperceptions)
   3. Local community dynamics— demographic, economic, political, history of land-use, migration, occupation
   4. Local environments (social, political, economic, occupational, physical, educational, public safety) and their impact on health
   5. History of practitioner-community relationships
   6. Isolation of the health care community from the community at large
   7. Relationship of formal and informal healthcare
10. Clinician-community relationship, domain approach, philosophy and attitudes
    1. Respect for community integrity, cultural diversity, and multiple determinants of health
    2. Understand health-relevant policy
    3. Is open-minded
    4. Is honest about the limits of medical care
    5. Appreciate responsibility to contribute health expertise to public dialogue
    6. Respect for community leadership
    7. Appreciate responsibility to work for the health of the public
11. Clinician-community relationship, domain behaviors
    1. Participate in community dialogue and development
    2. Participate in activities intended to ascertain the relationship between health care providers and community health, community health status, and the impact of health care delivery systems on community health
    3. Participate in the development of health enhancing community policy
    4. Communicate actively in matters of relevance to community health—listening openly, empowering others, contributing health expertise, facilitating the learning of others
    5. Participate actively in the implementation of community health strategies, health teams, and health care organizations
12. Clinician-community relationship, domain outcomes
    1. Enhanced collaboration between formal and informal health care ‘‘systems’’ within the local community
    2. Greater depth of understanding of the community’s health care resources, as well as vulnerabilities
    3. Greater prevalence of organizational policies that promote community health
    4. Greater participation of health care organizational personnel in civic service
    5. Enhanced community health

* Mary Catherine Beach, Thomas Inui, and the Relationship-Centered Care Research Network. Relationship-centered Care: A Constructive Reframing. Journal of General Internal Medicine. 2006; 21:S3–8. DOI: 10.1111/j.1525-1497.2006.00302.x

**Template version 2**

While analyzing data and with consensus in the research group, we identified new circumstances/situations that were related to relationships in healthcare and could not be attributed to elements described in literature. This was called ‘context’. With our aim to use quotations we already marked interesting textual elements. Elements that might be relevant for RCC, but seemed to be not that closely related to RCC were indicated as ‘other’. We continued analyzing data in small badges.

1. Clinician-patient, domain knowledge
   1. Each patient is a unique individual
   2. Psychosocial, emotional, and lifestyle issues are integral to medical care
   3. Patients differ in their values, preferences, and expectations for care
   4. Patients’ perspective, culture, and personality are relevant to the process of care
   5. "Each relationship is unique and is a product of the work of each participant"
   6. The manner in which a clinician participates in an encounter fundamentally affects the course, direction, and outcomes of care both episodically and longitudinally
2. Clinician-patient, domain approach, philosophy and attitudes
   1. "Value partnership with patients
   2. View patients as experts
   3. Acknowledge that patients deserve respect
   4. View the provider-patient relationship as a therapeutic vehicle
   5. Value the achievement of mutual respect and unconditional positive regard
   6. Acknowledge that affective engagement, rather than affective neutrality or detached concern, can further the therapeutic bond and its efficacy
   7. Acknowledge that clinicians and patients are both active human participants (not just role occupants) who co-construct their relationships
   8. Acknowledge that relationships are reciprocal and involve mutual tasks, duties an responsibilities
3. Clinician-patient, domain behaviors
   1. Show respect to patients
   2. Find out about patient’s values, expectations, preferences, and background
   3. Tailor approach to the patient based on knowledge of patient
   4. Help patient get story across, listen well (nonjudgmentally)
   5. Respond to patient’s emotions, show empathy
   6. Seek common ground as a point of departure for formulating therapeutic plans
   7. Attend to/monitor one’s own behavior as an influence on the other(s)
   8. Be aware of and acknowledge own feelings and biases (emotional self-awareness)
   9. Acknowledge the importance of relationships to the therapeutic process and outcome for both partners
   10. Acknowledge need to take both participants’ values, attitudes, and personality into account
   11. Acknowledge areas of agreement and disagreement on values, expectations, etc.
   12. Monitor the state of the relationship
   13. Acknowledge the importance of the relationship to one’s own well-being
4. Clinician-patient, domain outcomes
   1. Patient feels honored, respected, attended to, etc
   2. Patient likes and is satisfied with provider
   3. Patient has lower anxiety
   4. Patient has trust in provider
   5. Patient adheres to treatment
   6. Patient remembers information, advice
   7. Patient is more actively engaged
   8. Mutual attunement and harmony
   9. Informed decision making
   10. Added depth and vitality to interactions
   11. Clinician becomes a source of social and emotional support for the patient
   12. Patient becomes a source of professional reward/gratification for the clinician
   13. Protection against professional burnout
   14. Greater agreement on treatment plans
5. Clinician-clinician, domain knowledge
   1. Power inequities across health disciplines
   2. Power of understanding the other’s perspective
   3. Healing approaches of various health disciplines
   4. Team-building dynamics and approaches to shared leadership
6. Clinician-clinician relationship, domain approach, philosophy and attitudes
   1. Affirm importance of self awareness
   2. Value diversity and interdisciplinarity
   3. Appreciate importance of shared mission
   4. Is open to others’ ideas
   5. Affirm importance of, mutual respect, and trust
   6. Believe in importance of sustaining capacity for recognition, reconciliation, and prevention of error
7. Clinician-clinician relationship, domain behaviors
   1. Reflect on self and personal/ professional needs
   2. Continually learn from personal experience and that of others
   3. Learn cooperatively
   4. Derive personal meaning from the work of others
   5. Communicate effectively to other members of the team
   6. Listen actively to understand and engage other members of the team
   7. Work collaboratively, share responsibility
   8. Recognize and work to resolve conflicts
   9. Provide space in meetings for new thoughts, ideas
   10. Employ appreciative inquiry to imagine improvements
   11. Continuously examine whether the organizational values are reflected in day-to-day work
8. Clinician-clinician relationship, domain outcomes
   1. Productive resolution of disagreements
   2. Minimal staff turnover Improved ease of staff recruitment
   3. Colleagues reach personal and professional goals regularly
   4. Team members report being treated fairly and respectfully
   5. Enhanced capacity for working across a broad array of challenges
   6. Enhanced patient safety and quality of care
9. Clinician-community relationship, domain knowledge
   1. Diverse constructs/models of community
   2. Community perceptions of healthcare (including myths and misperceptions)
   3. Local community dynamics— demographic, economic, political, history of land-use, migration, occupation
   4. Local environments (social, political, economic, occupational, physical, educational, public safety) and their impact on health
   5. History of practitioner-community relationships
   6. Isolation of the health care community from the community at large
   7. Relationship of formal and informal healthcare
10. Clinician-community relationship, domain approach, philosophy and attitudes
    1. Respect for community integrity, cultural diversity, and multiple determinants of health
    2. Understand health-relevant policy
    3. Is open-minded
    4. Is honest about the limits of medical care
    5. Appreciate responsibility to contribute health expertise to public dialogue
    6. Respect for community leadership
    7. Appreciate responsibility to work for the health of the public
11. Clinician-community relationship, domain behaviors
    1. Participate in community dialogue and development
    2. Participate in activities intended to ascertain the relationship between health care providers and community health, community health status, and the impact of health care delivery systems on community health
    3. Participate in the development of health enhancing community policy
    4. Communicate actively in matters of relevance to community health—listening openly, empowering others, contributing health expertise, facilitating the learning of others
    5. Participate actively in the implementation of community health strategies, health teams, and health care organizations
12. Clinician-community relationship, domain outcomes
    1. Enhanced collaboration between formal and informal health care ‘‘systems’’ within the local community
    2. Greater depth of understanding of the community’s health care resources, as well as vulnerabilities
    3. Greater prevalence of organizational policies that promote community health
    4. Greater participation of health care organizational personnel in civic service
    5. Enhanced community health
13. **Other/relevant**
14. **Nice quote**
15. **Context**

**Template version 3 clarification**

Where possible and with consensus in our research group, themes that were closely related to each other were merged to one overarching theme. Themes absent in our data were deleted from the template. Both actions in order to make it a more comprehensive template. We continued analyzing data in small badges.

1. Clinician-patient, domain knowledge
   1. Unique personhood 🡪 (Each patient is a unique individual + Psychosocial, emotional, and lifestyle issues are integral to medical care + Patients differ in their values, preferences, and expectations for care + Patients’ perspective, culture, and personality are relevant to the process of care
   2. Each relationship is unique and is a product of the work of each participant
   3. The manner in which a clinician participates in an encounter fundamentally affects the course, direction, and outcomes of care both episodically and longitudinally
2. Clinician-patient, domain approach, philosophy and attitudes
   1. Respectful attitude towards patient and vice versa 🡪 (Value partnership with patients + View patients as experts + Acknowledge that patients deserve respect + Value the achievement of mutual respect and unconditional positive regard)
   2. Acknowledge the value of the relationship, the co-constructing role and mutual duties for all participants and its effect on the relationship 🡪 ( View the provider-patient relationship as a therapeutic vehicle + Acknowledge that affective engagement, rather than affective neutrality or detached concern, can further the therapeutic bond and its efficacy + Acknowledge that clinicians and patients are both active human participants (not just role occupants) who co-construct their relationships + Acknowledge that relationships are reciprocal and involve mutual tasks, duties and responsibilities
3. Clinician-patient, domain behaviors
   1. Show respect to patients
   2. Deepen the patient's context (values, attitudes, personality, expectations, preferences, background) by asking, listening 🡪 (Find out about patient’s values, expectations, preferences, and background + Help patient get story across, listen well (nonjudgmentally) + Acknowledge need to take both participants’ values, attitudes, and personality into account)
   3. Individualize approach 🡪 (Tailor approach to the patient based on knowledge of patient + Seek common ground as a point of departure for formulating therapeutic plans
   4. Respond to patient’s emotions, show empathy
   5. Own thoughts/self awareness and its effect on relationship 🡪 (Attend to/monitor one’s own behavior as an influence on the other(s) + Be aware of and acknowledge own feelings and biases (emotional self-awareness) + Acknowledge areas of agreement and disagreement on values, expectations, etc. + Acknowledge the importance of the relationship to one’s own well-being
   6. Acknowledge the importance of relationships to the therapeutic process and outcome for both partners
   7. DELETED 🡪Monitor the state of the relationship
4. Clinician-patient, domain outcomes
   1. Patient feels honored, respected, attended to, etc
   2. Patient likes and is satisfied with provider
   3. Patient has lower anxiety
   4. Patient has trust in provider
   5. Patient adheres to treatment
   6. Patient remembers information, advice
   7. Patient is more actively engaged
   8. Mutual attunement and harmony
   9. Informed decision making
   10. Added depth and vitality to interactions
   11. Clinician becomes a source of social and emotional support for the patient
   12. Patient becomes a source of professional reward/gratification for the clinician
   13. Protection against professional burnout
   14. Greater agreement on treatment plans
5. Clinician-clinician, domain knowledge
   1. Power inequities across health disciplines
   2. Power of understanding the other’s perspective
   3. Healing approaches of various health disciplines
   4. Team-building dynamics and approaches to shared leadership
6. Clinician-clinician relationship, domain approach, philosophy and attitudes
   1. Affirm importance of self awareness
   2. Value diversity and interdisciplinarity
   3. Appreciate importance of shared mission
   4. Is open to others’ ideas
   5. Affirm importance of, mutual respect, and trust
   6. Believe in importance of sustaining capacity for recognition, reconciliation, and prevention of error
7. Clinician-clinician relationship, domain behaviors
   1. Reflect on self and personal/ professional needs
   2. Continually learn from personal experience and that of others
   3. Learn cooperatively
   4. Derive personal meaning from the work of others
   5. Communicate effectively to other members of the team
   6. Listen actively to understand and engage other members of the team
   7. Work collaboratively, share responsibility
   8. Recognize and work to resolve conflicts
   9. Provide space in meetings for new thoughts, ideas
   10. Employ appreciative inquiry to imagine improvements
   11. Continuously examine whether the organizational values are reflected in day-to-day work
8. Clinician-clinician relationship, domain outcomes
   1. Productive resolution of disagreements
   2. Minimal staff turnover Improved ease of staff recruitment
   3. Colleagues reach personal and professional goals regularly
   4. Team members report being treated fairly and respectfully
   5. Enhanced capacity for working across a broad array of challenges
   6. Enhanced patient safety and quality of care
9. Clinician-community relationship, domain knowledge
   1. Diverse constructs/models of community
   2. Community perceptions of healthcare (including myths and misperceptions)
   3. Local community dynamics— demographic, economic, political, history of land-use, migration, occupation
   4. Local environments (social, political, economic, occupational, physical, educational, public safety) and their impact on health
   5. History of practitioner-community relationships
   6. Isolation of the health care community from the community at large
   7. Relationship of formal and informal healthcare
10. Clinician-community relationship, domain approach, philosophy and attitudes
    1. Respect for community integrity, cultural diversity, and multiple determinants of health
    2. Understand health-relevant policy
    3. Is open-minded
    4. Is honest about the limits of medical care
    5. Appreciate responsibility to contribute health expertise to public dialogue
    6. Respect for community leadership
    7. Appreciate responsibility to work for the health of the public
11. Clinician-community relationship, domain behaviors
    1. Participate in community dialogue and development
    2. Participate in activities intended to ascertain the relationship between health care providers and community health, community health status, and the impact of health care delivery systems on community health
    3. Participate in the development of health enhancing community policy
    4. Communicate actively in matters of relevance to community health—listening openly, empowering others, contributing health expertise, facilitating the learning of others
    5. Participate actively in the implementation of community health strategies, health teams, and health care organizations
12. Clinician-community relationship, domain outcomes
    1. Enhanced collaboration between formal and informal health care ‘‘systems’’ within the local community
    2. Greater depth of understanding of the community’s health care resources, as well as vulnerabilities
    3. Greater prevalence of organizational policies that promote community health
    4. Greater participation of health care organizational personnel in civic service
    5. Enhanced community health
13. Other/relevant
14. Nice quote
15. Context

**Template version 3, overview**

1. Clinician-patient, domain knowledge
   1. Unique personhood
   2. Each relationship is unique and is a product of the work of each participant
   3. The manner in which a clinician participates in an encounter fundamentally affects the course, direction, and outcomes of care both episodically and longitudinally
2. Clinician-patient, domain approach, philosophy and attitudes
   1. Respectful attitude towards patient and vice versa
   2. Acknowledge the value of the relationship, the co-constructing role and mutual duties for all participants and its effect on the relationship
3. Clinician-patient, domain behaviors
   1. Show respect to patients
   2. Deepen the patient's context
   3. Individualize approach
   4. Respond to patient’s emotions, show empathy
   5. Own thoughts/self awareness and its effect on relationship
   6. Acknowledge the importance of relationships to the therapeutic process and outcome for both partners
4. Clinician-patient, domain outcomes
   1. Patient feels honored, respected, attended to, etc
   2. Patient likes and is satisfied with provider
   3. Patient has lower anxiety
   4. Patient has trust in provider
   5. Patient adheres to treatment
   6. Patient remembers information, advice
   7. Patient is more actively engaged
   8. Mutual attunement and harmony
   9. Informed decision making
   10. Added depth and vitality to interactions
   11. Clinician becomes a source of social and emotional support for the patient
   12. Patient becomes a source of professional reward/gratification for the clinician
   13. Protection against professional burnout
   14. Greater agreement on treatment plans
5. Clinician-clinician, domain knowledge
   1. Power inequities across health disciplines
   2. Power of understanding the other’s perspective
   3. Healing approaches of various health disciplines
   4. Team-building dynamics and approaches to shared leadership
6. Clinician-clinician relationship, domain approach, philosophy and attitudes
   1. Affirm importance of self awareness
   2. Value diversity and interdisciplinarity
   3. Appreciate importance of shared mission
   4. Is open to others’ ideas
   5. Affirm importance of, mutual respect, and trust
   6. Believe in importance of sustaining capacity for recognition, reconciliation, and prevention of error
7. Clinician-clinician relationship, domain behaviors
   1. Reflect on self and personal/ professional needs
   2. Continually learn from personal experience and that of others
   3. Learn cooperatively
   4. Derive personal meaning from the work of others
   5. Communicate effectively to other members of the team
   6. Listen actively to understand and engage other members of the team
   7. Work collaboratively, share responsibility
   8. Recognize and work to resolve conflicts
   9. Provide space in meetings for new thoughts, ideas
   10. Employ appreciative inquiry to imagine improvements
   11. Continuously examine whether the organizational values are reflected in day-to-day work
8. Clinician-clinician relationship, domain outcomes
   1. Productive resolution of disagreements
   2. Minimal staff turnover Improved ease of staff recruitment
   3. Colleagues reach personal and professional goals regularly
   4. Team members report being treated fairly and respectfully
   5. Enhanced capacity for working across a broad array of challenges
   6. Enhanced patient safety and quality of care
9. Clinician-community relationship, domain knowledge
   1. Diverse constructs/models of community
   2. Community perceptions of healthcare (including myths and misperceptions)
   3. Local community dynamics— demographic, economic, political, history of land-use, migration, occupation
   4. Local environments (social, political, economic, occupational, physical, educational, public safety) and their impact on health
   5. History of practitioner-community relationships
   6. Isolation of the health care community from the community at large
   7. Relationship of formal and informal healthcare
10. Clinician-community relationship, domain approach, philosophy and attitudes
    1. Respect for community integrity, cultural diversity, and multiple determinants of health
    2. Understand health-relevant policy
    3. Is open-minded
    4. Is honest about the limits of medical care
    5. Appreciate responsibility to contribute health expertise to public dialogue
    6. Respect for community leadership
    7. Appreciate responsibility to work for the health of the public
11. Clinician-community relationship, domain behaviors
    1. Participate in community dialogue and development
    2. Participate in activities intended to ascertain the relationship between health care providers and community health, community health status, and the impact of health care delivery systems on community health
    3. Participate in the development of health enhancing community policy
    4. Communicate actively in matters of relevance to community health—listening openly, empowering others, contributing health expertise, facilitating the learning of others
    5. Participate actively in the implementation of community health strategies, health teams, and health care organizations
12. Clinician-community relationship, domain outcomes
    1. Enhanced collaboration between formal and informal health care ‘‘systems’’ within the local community
    2. Greater depth of understanding of the community’s health care resources, as well as vulnerabilities
    3. Greater prevalence of organizational policies that promote community health
    4. Greater participation of health care organizational personnel in civic service
    5. Enhanced community health
13. Other/relevant
14. Nice quote
15. Context

**Template version 4, clarification**

By further analyzing our data more themes could be merged and renamed. Other themes were deleted. All in consensus with the research group. The theme ‘other/relevant’ could be deleted, either allocating these themes to already existing theme, or considering these themes to be irrelevant or not suitable to answer the research question. The theme context was further divided into context-human and context non-human, as relevant contributors in relationship centredness. We continued analyzing data in small badges.

1. Clinician-patient, domain knowledge
   1. Unique personhood
   2. Each relationship is unique and is a product of the work of each participant
   3. The manner in which a clinician participates in an encounter fundamentally affects the course, direction, and outcomes of care both episodically and longitudinally
2. Clinician-patient, domain approach, philosophy and attitudes
   1. Respectful attitude towards patient and vice versa
   2. Acknowledge the value of the relationship, the co-constructing role and mutual duties for all participants and its effect on the relationship
3. Clinician-patient, domain behaviors
   1. Show respect to patients
   2. Deepen the patient's context
   3. Individualize approach
   4. Respond to patient’s emotions, show empathy
   5. Own thoughts/self awareness and its effect on relationship
   6. Acknowledge the importance of relationships to the therapeutic process and outcome for both partners
4. Clinician-patient, domain outcomes
   1. Outcome feeling 🡪 (Patient feels honored, respected, attended to, etc + Patient likes and is satisfied with provider + Patient has lower anxiety + Patient has trust in provider
   2. Outcome action 🡪 (Patient adheres to treatment + Patient remembers information, advice + Patient is more actively engaged + Greater agreement on treatment plans
   3. Outcome interaction 🡪 (Mutual attunement and harmony + Informed decision making + Added depth and vitality to interactions + Clinician becomes a source of social and emotional support for the patient + Patient becomes a source of professional reward/gratification for the clinician)
   4. DELETED 🡪 Protection against professional burnout
5. Clinician-clinician, domain knowledge
   1. Be aware of the importance/power of other health disciplines 🡪 (Power of understanding the other’s perspective + Healing approaches of various health disciplines)
   2. DELETED 🡪 Power inequities across health disciplines
   3. DELETED 🡪 Team-building dynamics and approaches to shared leadership
6. Clinician-clinician relationship, domain approach, philosophy and attitudes
   1. Value other health disciplines to help (Value diversity and interdisciplinarity + Appreciate importance of shared mission + Is open to others’ ideas + Affirm importance of, mutual respect, and trust
   2. DELETED 🡪 Affirm importance of self awareness
   3. DELETED 🡪 Believe in importance of sustaining capacity for recognition, reconciliation, and prevention of error
7. Clinician-clinician relationship, domain behaviors
   1. Work together efficiently with other members of the team (Communicate effectively to other members of the team + Listen actively to understand and engage other members of the team + Work collaboratively, share responsibility )
   2. DELETED 🡪 Reflect on self and personal/ professional needs
   3. DELETED 🡪 Continually learn from personal experience and that of others
   4. DELETED 🡪 Learn cooperatively
   5. DELETED 🡪 Derive personal meaning from the work of others
   6. DELETED 🡪Recognize and work to resolve conflicts
   7. DELETED 🡪 Provide space in meetings for new thoughts, ideas
   8. DELETED 🡪 Employ appreciative inquiry to imagine improvements
   9. DELETED 🡪 Continuously examine whether the organizational values are reflected in day-to-day work
8. Clinician-clinician relationship, domain outcomes
   1. DELETED 🡪 Productive resolution of disagreements
   2. DELETED 🡪 Minimal staff turnover Improved ease of staff recruitment
   3. DELETED 🡪 Colleagues reach personal and professional goals regularly
   4. DELETED 🡪 Team members report being treated fairly and respectfully
   5. DELETED 🡪 Enhanced capacity for working across a broad array of challenges
   6. DELETED 🡪 Enhanced patient safety and quality of care
9. Clinician-community relationship, domain knowledge
   1. Community perceptions 🡪 Diverse constructs/models of community +

Community perceptions of healthcare (including myths and misperceptions)

- 1. Community context 🡪 Local community dynamics— demographic, economic, political, history of land-use, migration, occupation + Local environments (social, political, economic, occupational, physical, educational, public safety) and their impact on health
  2. DELETED 🡪 History of practitioner-community relationships
  3. DELETED 🡪 Isolation of the health care community from the community at large
  4. DELETED 🡪 Relationship of formal and informal healthcare

1. Clinician-community relationship, domain approach, philosophy and attitudes
   1. Open minded 🡪 Respect for community integrity, cultural diversity, and multiple determinants of health + Is open-minded
   2. Is honest about the limits of medical care
   3. DELETED 🡪 Understand health-relevant policy
   4. DELETED 🡪 Appreciate responsibility to contribute health expertise to public dialogue
   5. DELETED 🡪 Respect for community leadership
   6. DELETED 🡪 Appreciate responsibility to work for the health of the public
2. Clinician-community relationship, domain behaviors
   1. Participate in community dialogue and development
   2. Participate in activities intended to ascertain the relationship between health care providers and community health, community health status, and the impact of health care delivery systems on community health
   3. DELETED 🡪 Participate in the development of health enhancing community policy
   4. DELETED 🡪 Communicate actively in matters of relevance to community health—listening openly, empowering others, contributing health expertise, facilitating the learning of others
   5. DELETED 🡪 Participate actively in the implementation of community health strategies, health teams, and health care organizations
3. Clinician-community relationship, domain outcomes
   1. DELETED 🡪 Enhanced collaboration between formal and informal health care ‘‘systems’’ within the local community
   2. DELETED 🡪 Greater depth of understanding of the community’s health care resources, as well as vulnerabilities
   3. DELETED 🡪 Greater prevalence of organizational policies that promote community health
   4. DELETED 🡪 Greater participation of health care organizational personnel in civic service
   5. DELETED 🡪 Enhanced community health
4. DELETED 🡪 Other/relevant
5. Nice quote
6. Context 🡪 subdivided in context human (Context patient relatives: impact disease on relatives or impact relatives on health behaviour patient or health care in general) and context non-human (Context patient social environment: impact disease on social environment (non-human) or impact social environment (non-human) on health care behaviour or health care in general)

**Template version 4, overview**

1. Clinician-patient, domain knowledge
   1. Unique personhood
   2. Each relationship is unique and is a product of the work of each participant
   3. The manner in which a clinician participates in an encounter fundamentally affects the course, direction, and outcomes of care both episodically and longitudinally
2. Clinician-patient, domain approach, philosophy and attitudes
   1. Respectful attitude towards patient and vice versa
   2. Acknowledge the value of the relationship, the co-constructing role and mutual duties for all participants and its effect on the relationship
3. Clinician-patient, domain behaviors
   1. Show respect to patients
   2. Deepen the patient's context
   3. Individualize approach
   4. Respond to patient’s emotions, show empathy
   5. Own thoughts/self awareness and its effect on relationship
   6. Acknowledge the importance of relationships to the therapeutic process and outcome for both partners
4. Clinician-patient, domain outcomes
   1. Outcome feeling
   2. Outcome action
   3. Outcome interaction
5. Clinician-clinician, domain knowledge
   1. Be aware of the importance/power of other health disciplines
6. Clinician-clinician relationship, domain approach, philosophy and attitudes
   1. Value other health disciplines to help
7. Clinician-clinician relationship, domain behaviors
   1. Work together efficiently with other members of the team
8. Clinician-community relationship, domain knowledge
   1. Community perceptions
   2. Community context
9. Clinician-community relationship, domain approach, philosophy and attitudes
   1. Open minded
   2. Is honest about limits of health care
10. Clinician-community relationship, domain behavior
    1. Participate in community dialogue and development
    2. Participate in activities intended to ascertain the relationship between health care providers and community health, community health status, and the impact of health care delivery systems on community health
11. Nice quote
12. Context human
13. Context non-human

**Template, version 5: clarification**

After consensus in the research group one new theme: ‘perspectives on future role/work field was added. We used this final template and performed analysis of all previous reports in an iterative process. We continued analyzing data in small badges until the research group reached consensus about data saturation.

1. Clinician-patient, domain knowledge
   1. Unique personhood
   2. Each relationship is unique and is a product of the work of each participant
   3. The manner in which a clinician participates in an encounter fundamentally affects the course, direction, and outcomes of care both episodically and longitudinally
2. Clinician-patient, domain approach, philosophy and attitudes
   1. Respectful attitude towards patient and vice versa
   2. Acknowledge the value of the relationship, the co-constructing role and mutual duties for all participants and its effect on the relationship
3. Clinician-patient, domain behaviors
   1. Show respect to patients
   2. Deepen the patient's context
   3. Individualize approach
   4. Respond to patient’s emotions, show empathy
   5. Own thoughts/self awareness and its effect on relationship
   6. Acknowledge the importance of relationships to the therapeutic process and outcome for both partners
4. Clinician-patient, domain outcomes
   1. Outcome feeling
   2. Outcome action
   3. Outcome interaction
5. Clinician-clinician, domain knowledge
   1. Be aware of the importance/power of other health disciplines
6. Clinician-clinician relationship, domain approach, philosophy and attitudes
   1. Value other health disciplines to help
7. Clinician-clinician relationship, domain behaviors
   1. Work together efficiently with other members of the team
8. Clinician-community relationship, domain knowledge
   1. Community perceptions
   2. Perspectives on future role/workfield (a student learns about how from a patients perspective the future role, profession or workfield is experienced.
   3. Community context
9. Clinician-community relationship, domain approach, philosophy and attitudes
   1. Open minded
   2. Is honest about limits of health care
10. Clinician-community relationship, domain behavior
    1. Participate in community dialogue and development
    2. Participate in activities intended to ascertain the relationship between health care providers and community health, community health status, and the impact of health care delivery systems on community health
11. Nice quote
12. Context human
13. Context non-human

**Template, version 5, overview, final version**

1. Clinician-patient, domain knowledge
   1. Unique personhood
   2. Each relationship is unique and is a product of the work of each participant
   3. The manner in which a clinician participates in an encounter fundamentally affects the course, direction, and outcomes of care both episodically and longitudinally
2. Clinician-patient, domain approach, philosophy and attitudes
   1. Respectful attitude towards patient and vice versa
   2. Acknowledge the value of the relationship, the co-constructing role and mutual duties for all participants and its effect on the relationship
3. Clinician-patient, domain behaviors
   1. Show respect to patients
   2. Deepen the patient's context
   3. Individualize approach
   4. Respond to patient’s emotions, show empathy
   5. Own thoughts/self awareness and its effect on relationship
   6. Acknowledge the importance of relationships to the therapeutic process and outcome for both partners
4. Clinician-patient, domain outcomes
   1. Outcome feeling
   2. Outcome action
   3. Outcome interaction
5. Clinician-clinician, domain knowledge
   1. Be aware of the importance/power of other health disciplines
6. Clinician-clinician relationship, domain approach, philosophy and attitudes
   1. Value other health disciplines to help
7. Clinician-clinician relationship, domain behaviors
   1. Work together efficiently with other members of the team
8. Clinician-community relationship, domain knowledge
   1. Community perceptions
   2. Perspectives on future role/workfield
   3. Community context
9. Clinician-community relationship, domain approach, philosophy and attitudes
   1. Open minded
   2. Is honest about the limits of healthcare
10. Clinician-community relationship, domain behavior
    1. Participate in community dialogue and development
    2. Participate in activities intended to ascertain the relationship between health care providers and community health, community health status, and the impact of health care delivery systems on community health
11. Nice quote
12. Context human
13. Context non-human
