# Supplementary material for: A Longitudinal Early Patient Encounter Program Through a Lens of Relationship-Centered Care
Source: J Gen Intern Med. 2025 Aug 12;40(16):3862–9. doi: 10.1007/s11606-025-09666-5 (PMC12686266; doi:10.1007/s11606-025-09666-5)
Supplement: Supplementary file 2 — (DOCX 79.6 KB) [file 11606_2025_9666_MOESM2_ESM.docx]

| **RCC themes** | **Knowledge** | **Skills** | **Attitude** | **Behavior** |
| --- | --- | --- | --- | --- |
| 1. Every patient has a unique personhood: psychosocial/emotional/lifestyle factors, values/ preferences/expectations for care, perspective/culture/personality. | 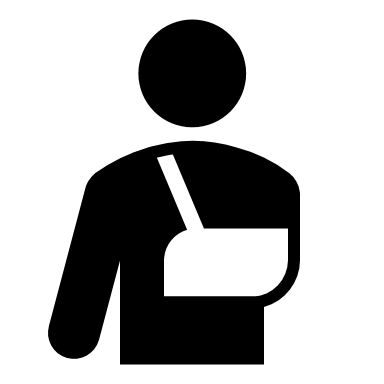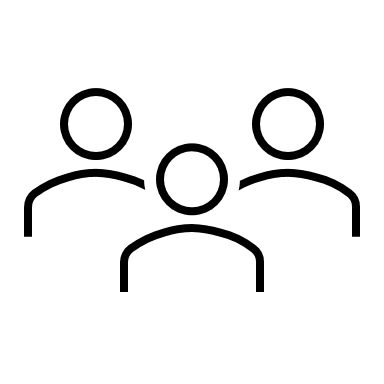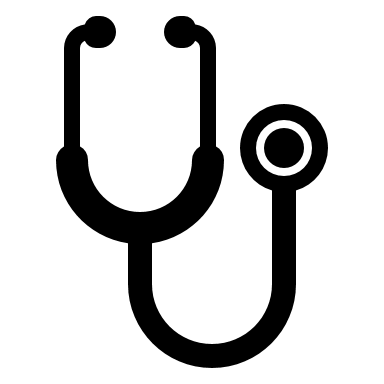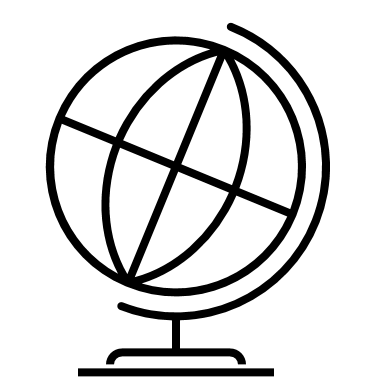 | 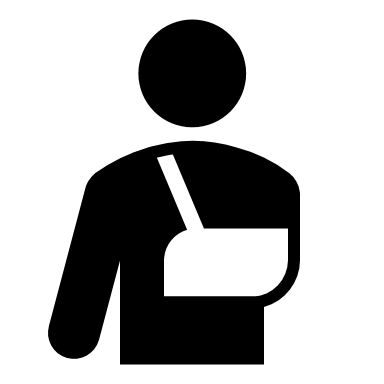 | 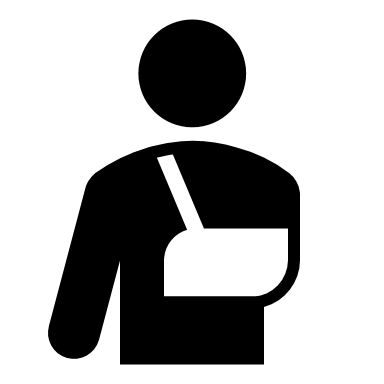 | 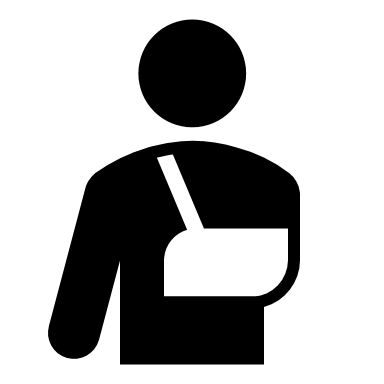 |
| 2. Each relationship is unique and is a product of the work of each participant. | 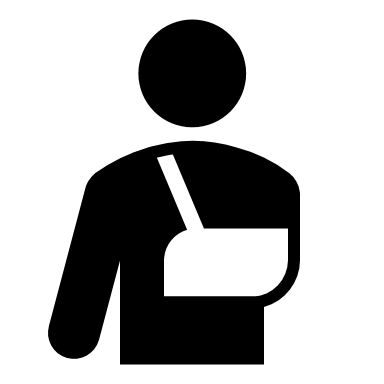 | 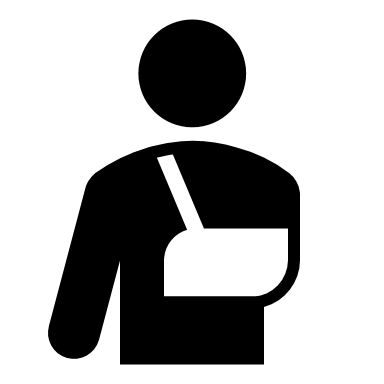 | 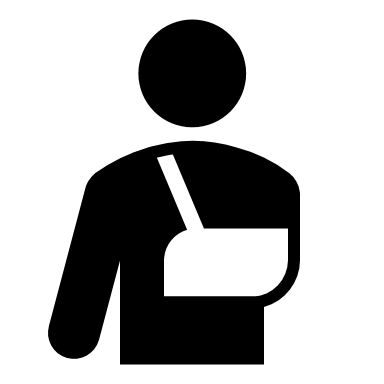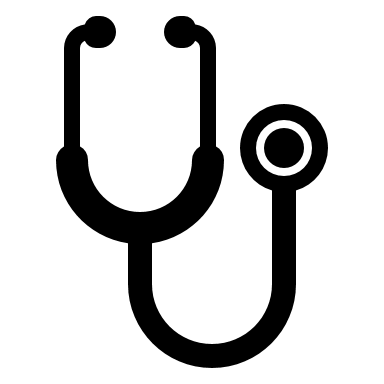 |  |
| 3. Acknowledging the value of the relationship, the co-constructing role and mutual duties for all participants and its effect on the relationship. | 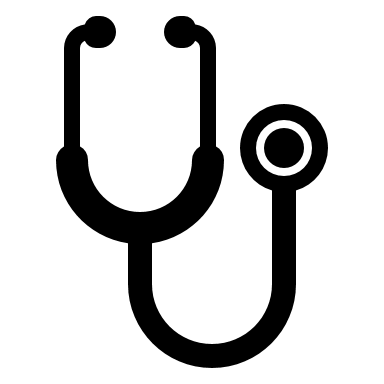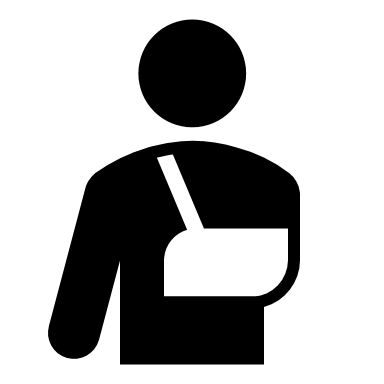 |  | 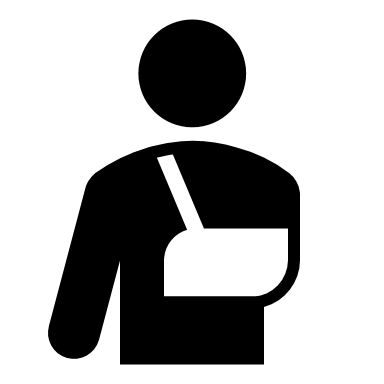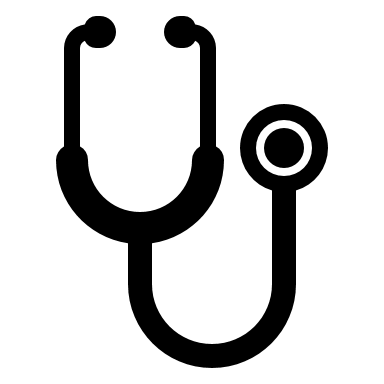 |  |
| 4. Deepening the patient's context (values, attitudes, personality, expectations, preferences, background). | 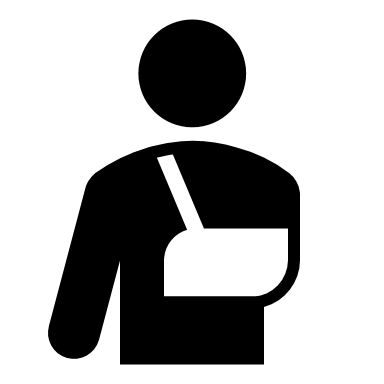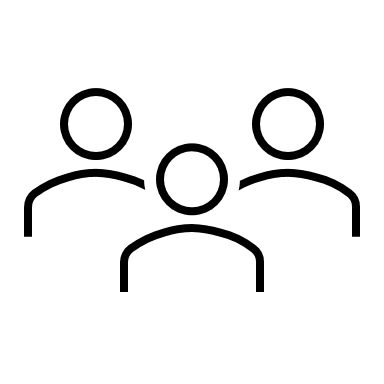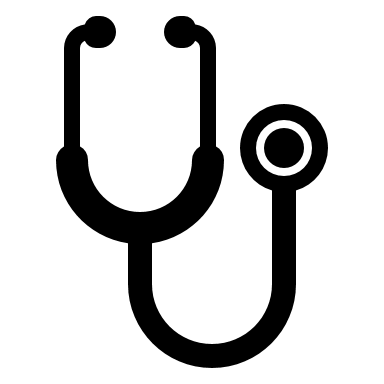 | 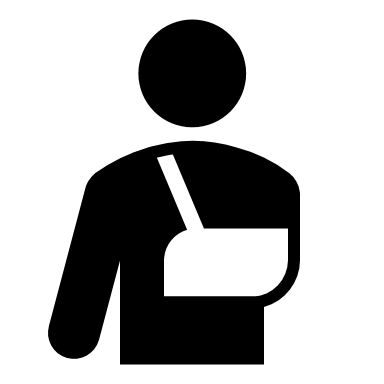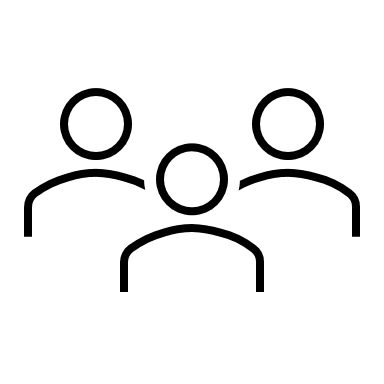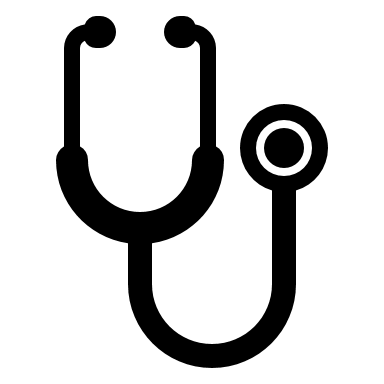 | 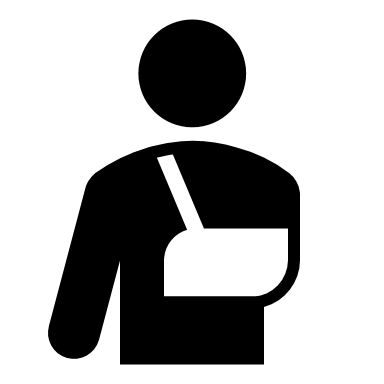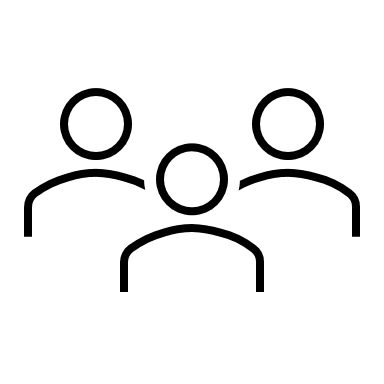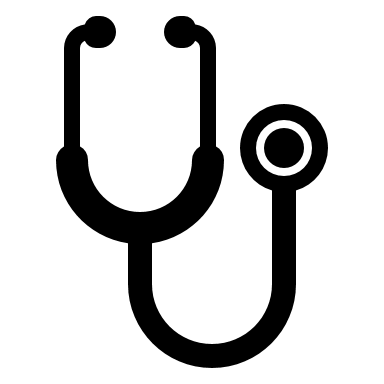 | 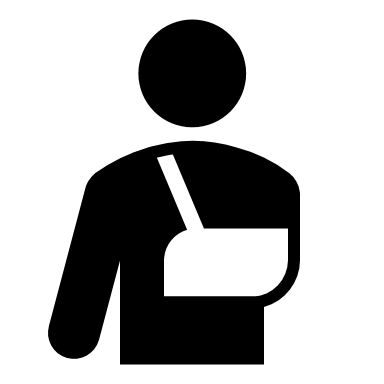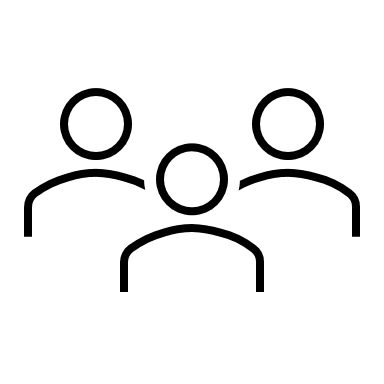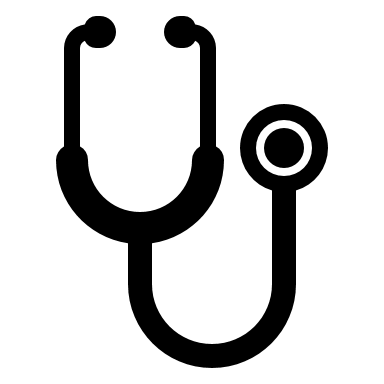 |
| 5. Respect in healthcare relations. | 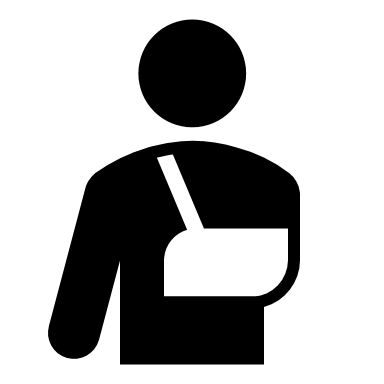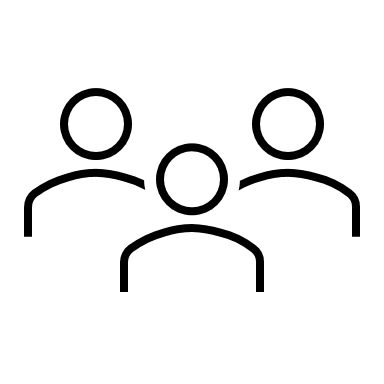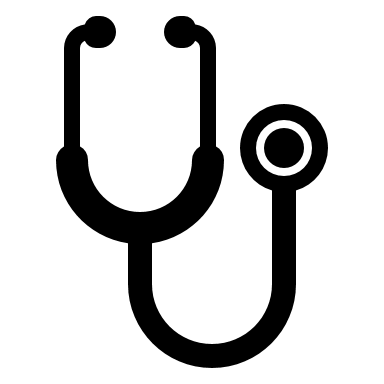 | 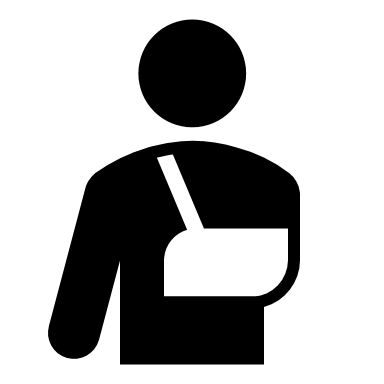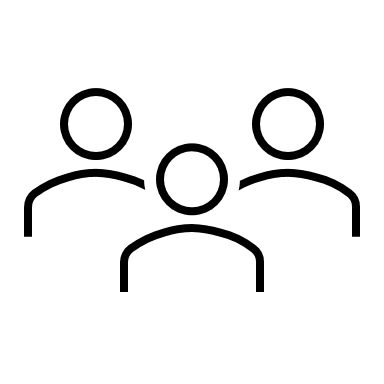 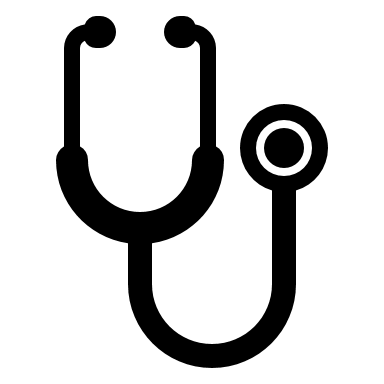 | 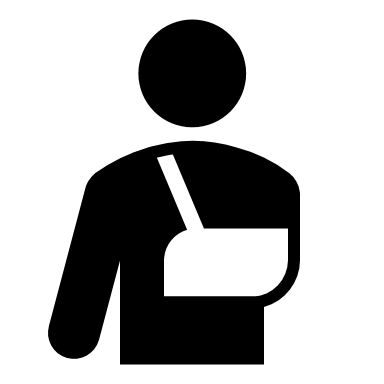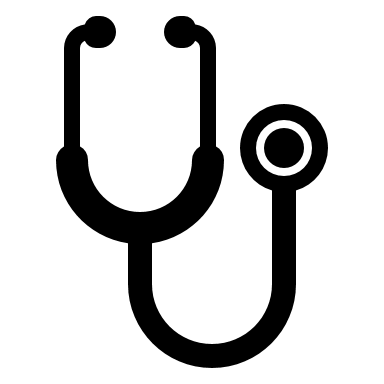 | 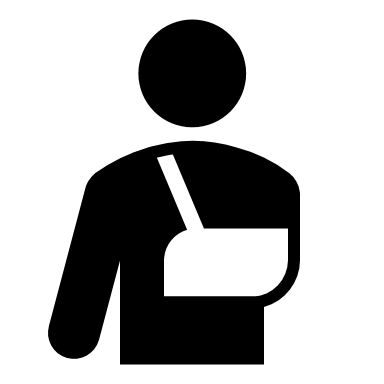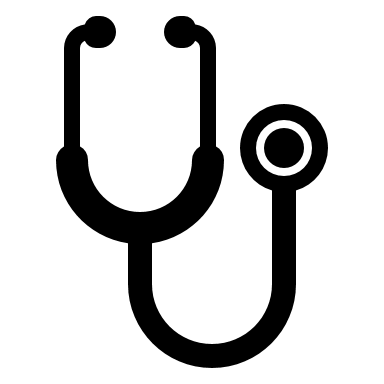 |
| 6. Empathy. | 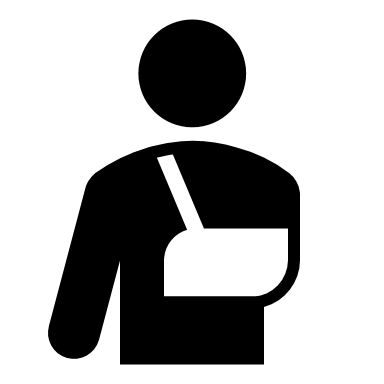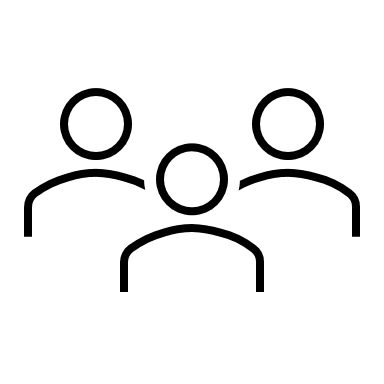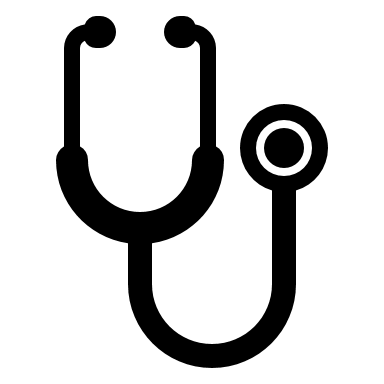 | 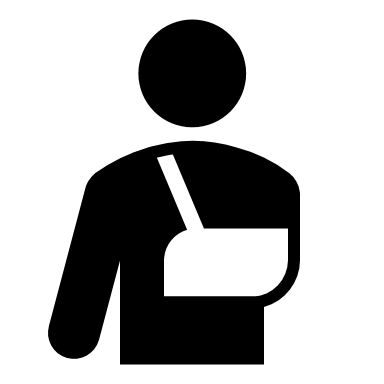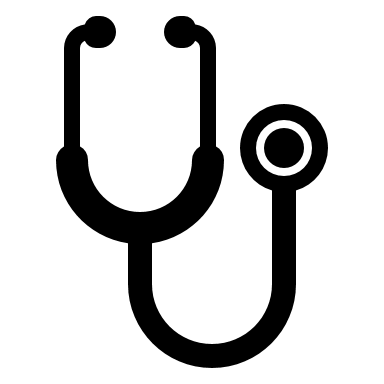 | 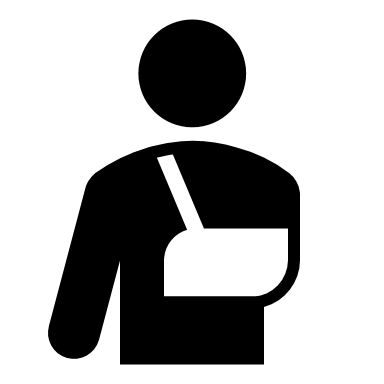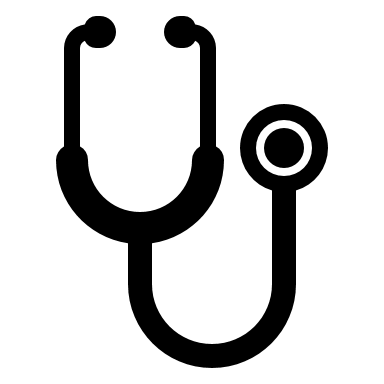 | 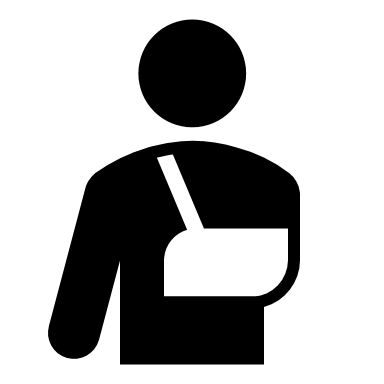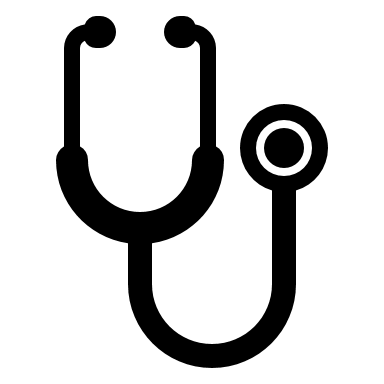 |
| 7. Manner of participation and its effect on healthcare relations. | 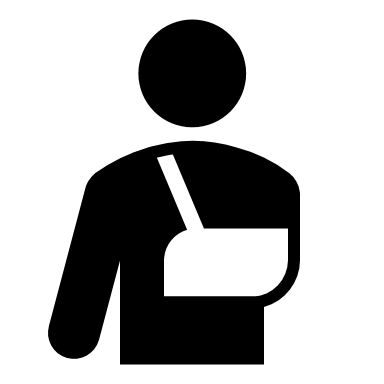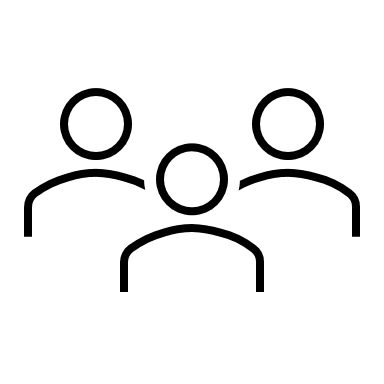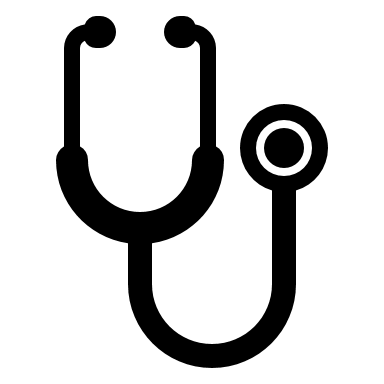 | 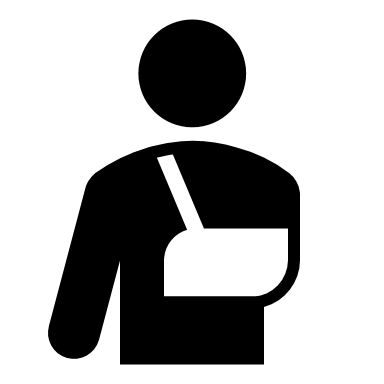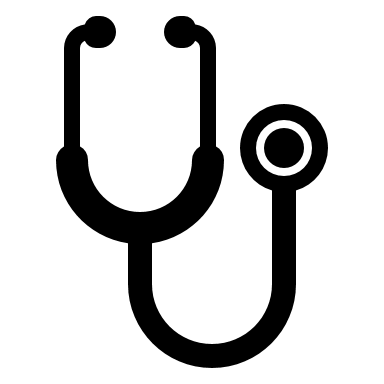 | 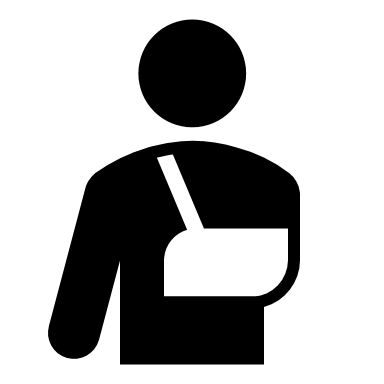 | 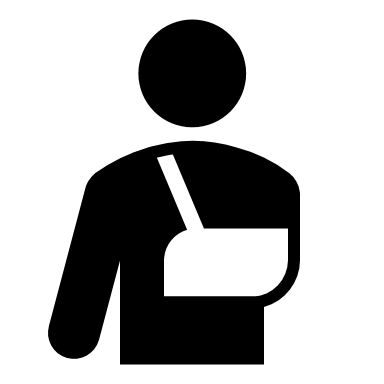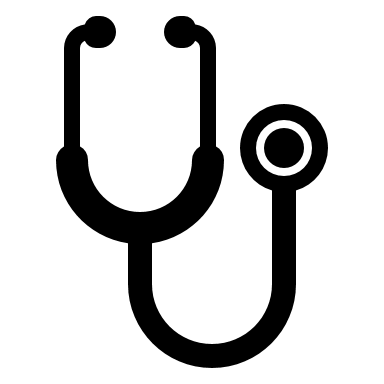 |
| 8. Being open minded. | 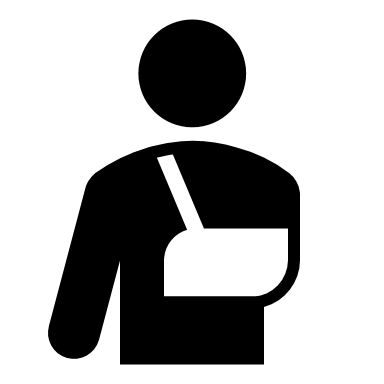 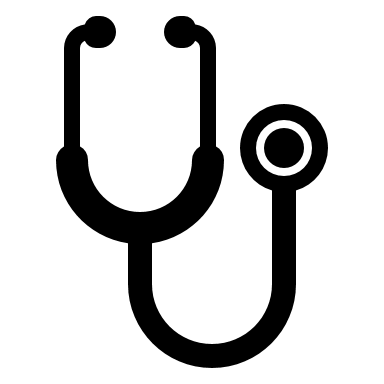 |  | 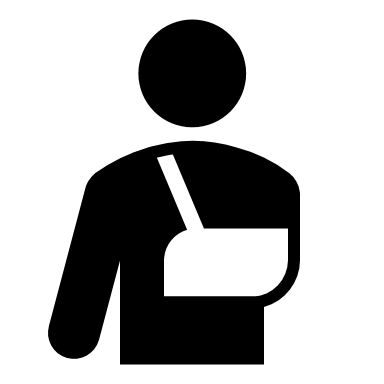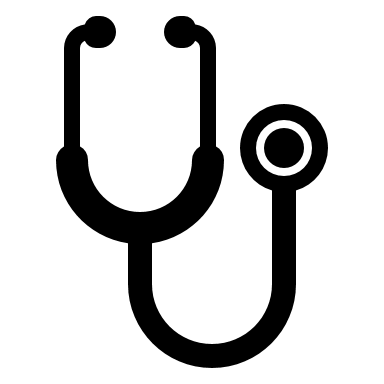 |  |
| 9. Individualizing approach in healthcare. | 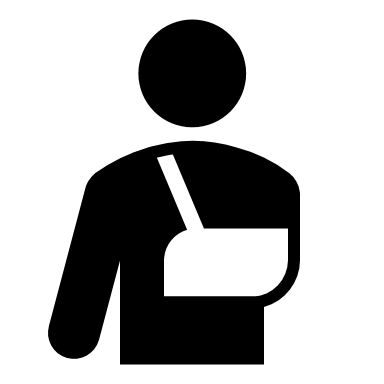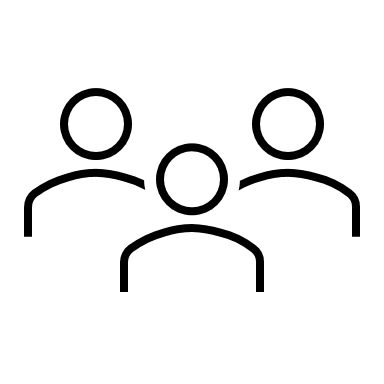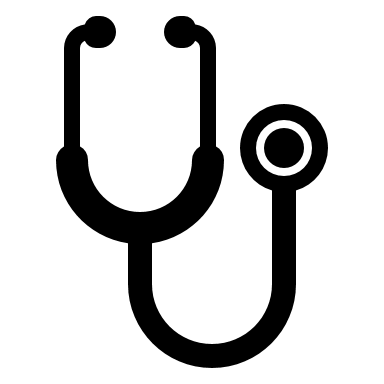 | 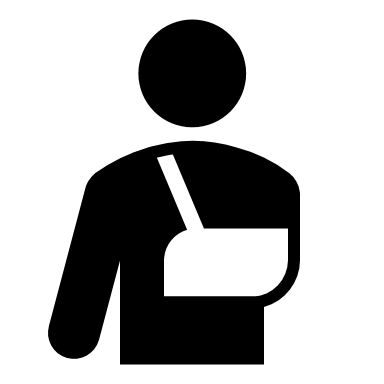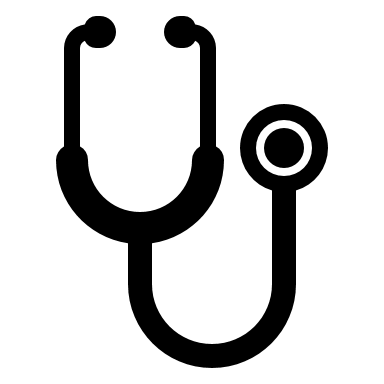 | 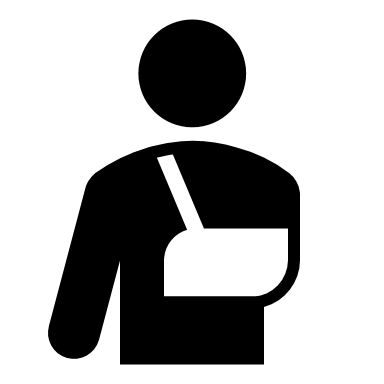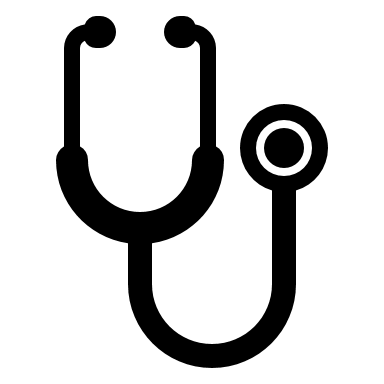 | 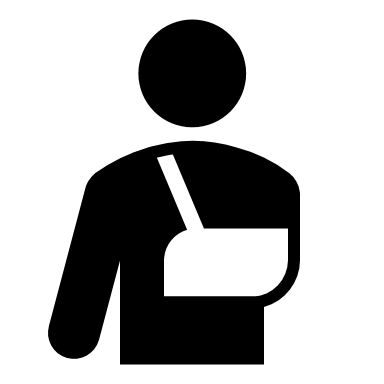 |
| 10. Being honest about limits of healthcare. | 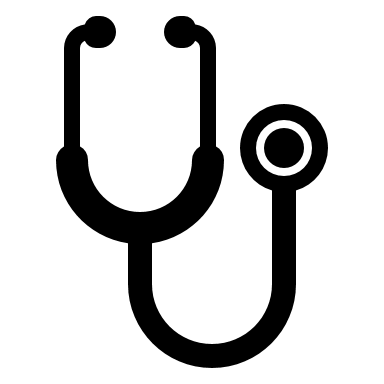 | 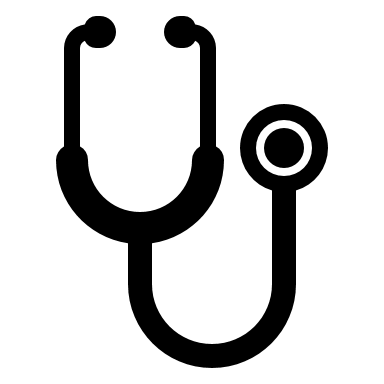 | 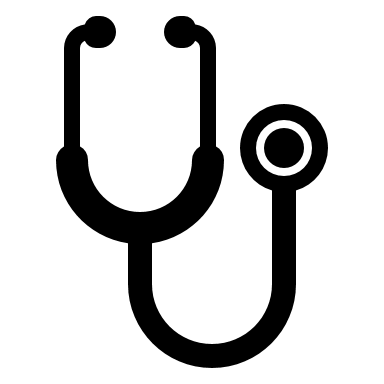 |  |
| 11. Outcome patient to treatment: adherence, active engagement. | 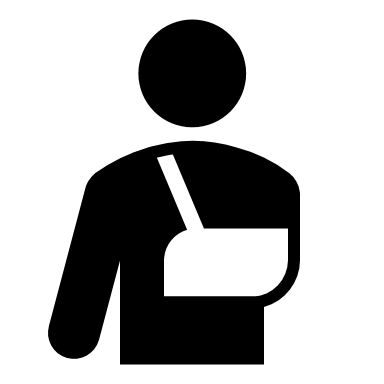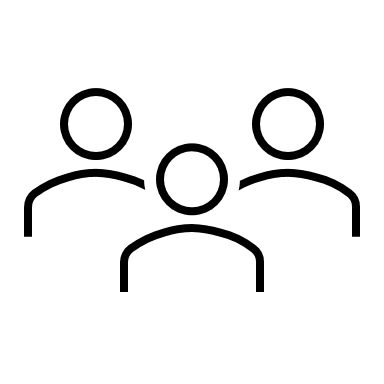 |  | 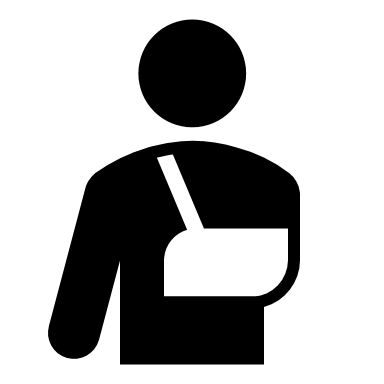 |  |
| 12. Impact healthcare relations on interaction with patient. | 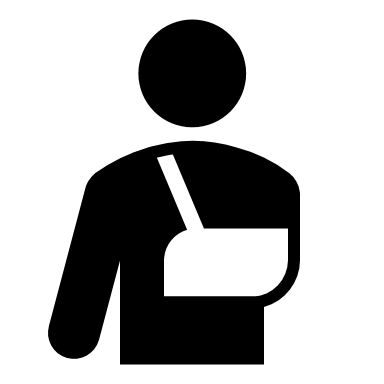 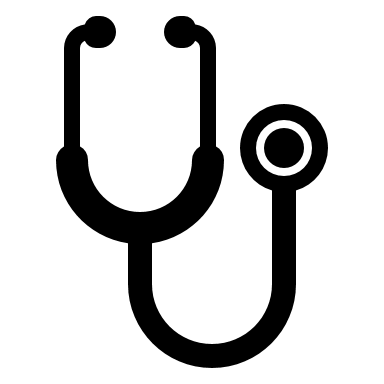 | 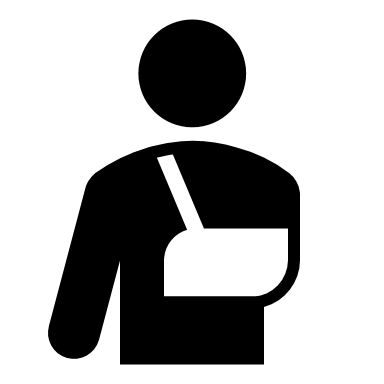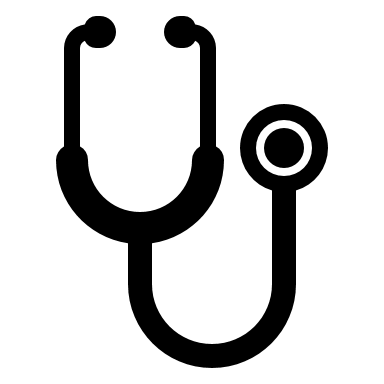 | 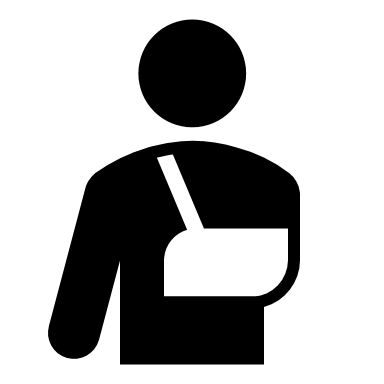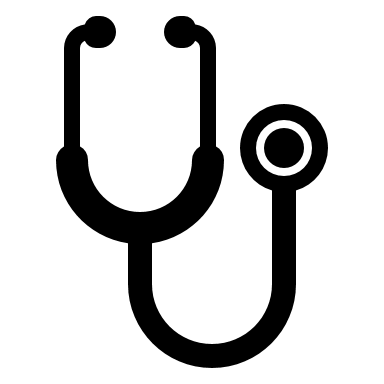 | 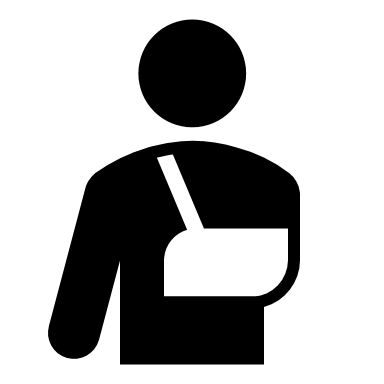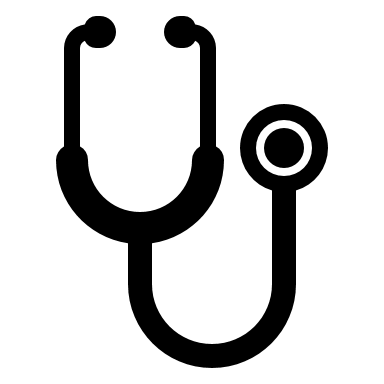 |
| 13. Impact healthcare relations on feelings patient. | 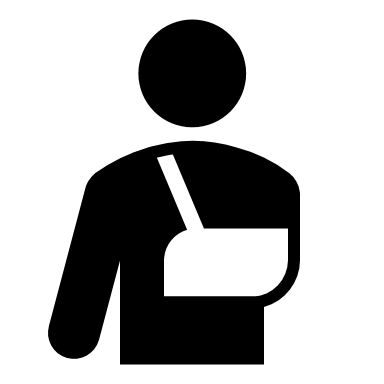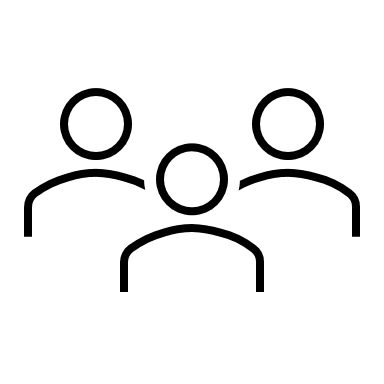 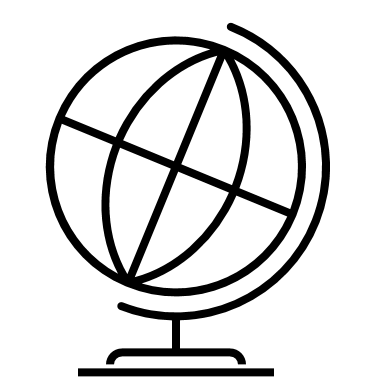 |  |  |  |
| 14. Impact healthcare relations on health/disease/behavior of patient. | 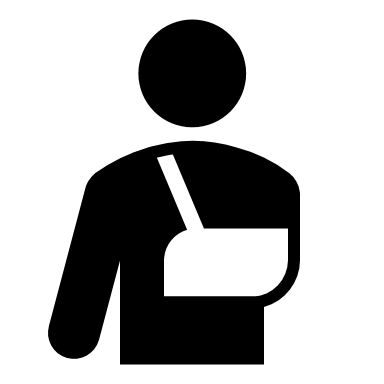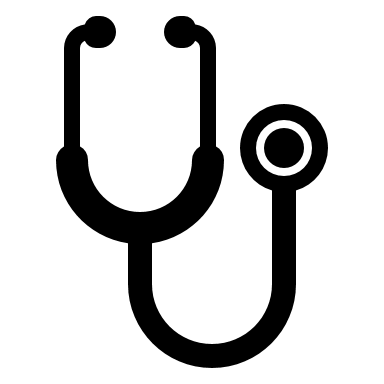 | 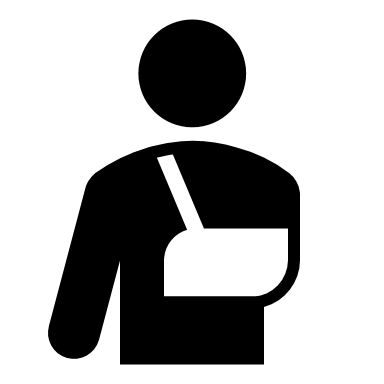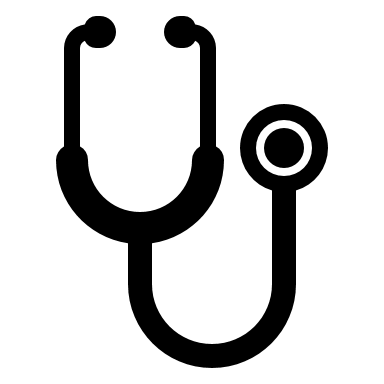 | 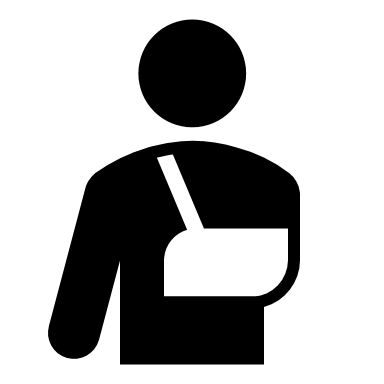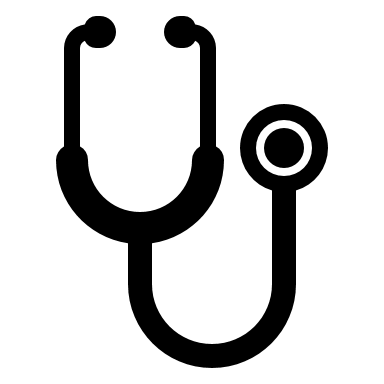 | 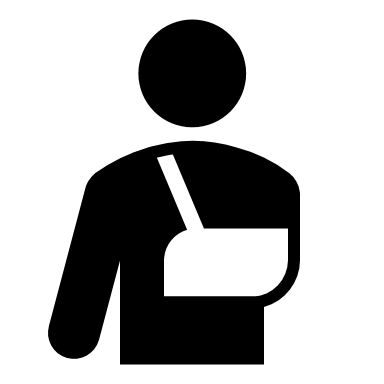 |
| 15. Being aware of the importance/power of other health disciplines. | 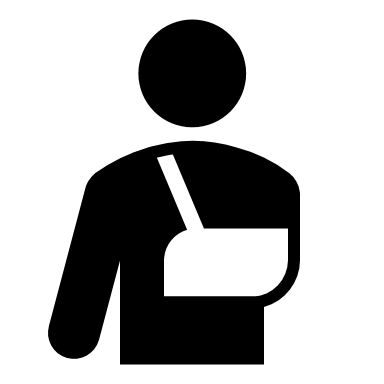 |  |  |  |
| 16. Work together efficiently with other members of the team. | 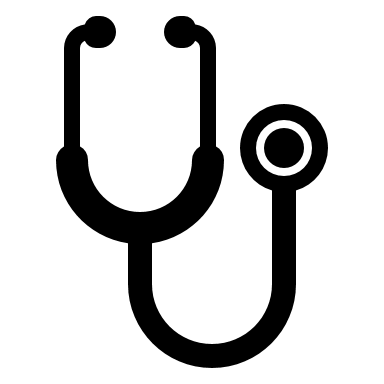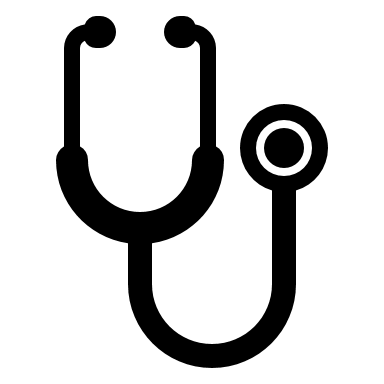 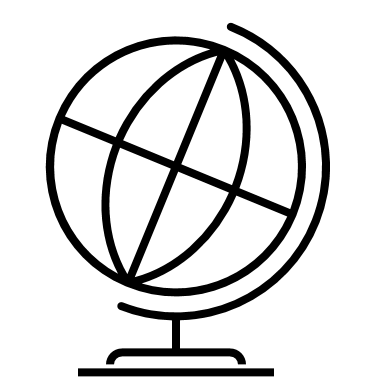 |  | 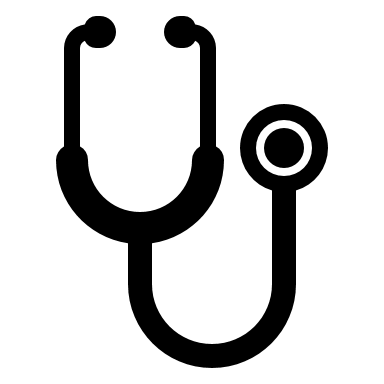 |  |
| 17. Recognize and work to resolve conflicts. | 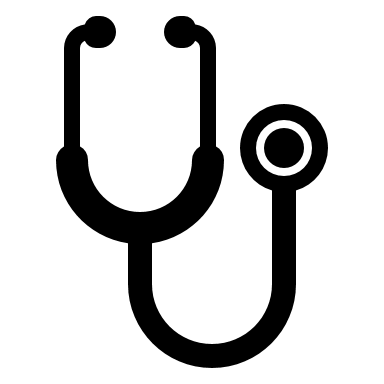 |  |  |  |
| 18. Communicate effectively in matters of relevance to community health. | 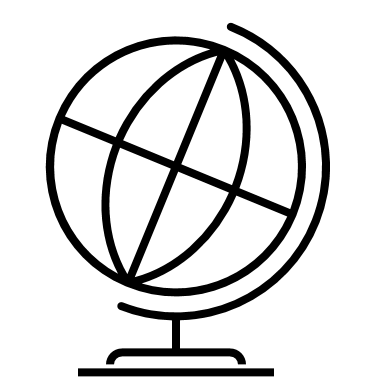 |  |  |  |
| 19. Participate in community dialogue and development. | 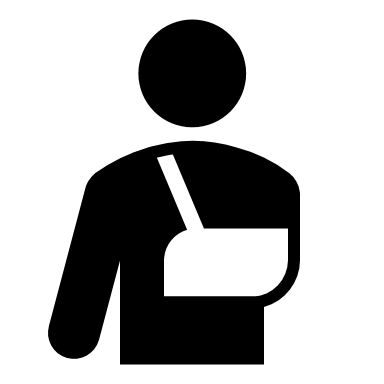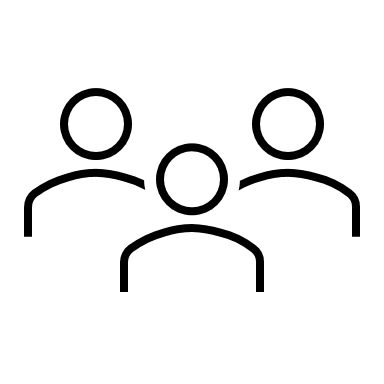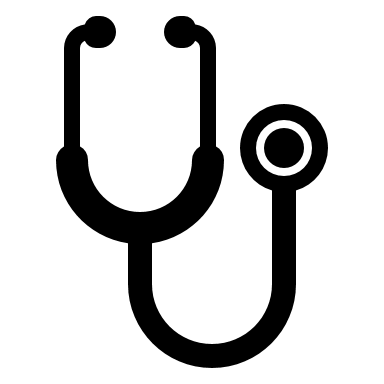 |  |  |  |
| 20. Community perceptions of healthcare. |  |  |  |  |
| 21. Community context and impact on healthcare. |  |  |  |  |
| 22. Self-awareness about relations in healthcare. |  |  |  |  |
| 23. Perspectives on future role/work field. |  |  |  |  |
| 24. Impact disease on relatives or impact relatives on health behavior patient or healthcare in general. |  |  |  |  |
| 25. Impact disease on social environment (non-human) or impact social environment (non-human) on health care behavior or healthcare in general. |  |  |  |  |

*Overview of the different RCC themes students’ described learning experiences (left vertical column), whether data identified themes of knowledge, skills, attitude or competence and from which participant in healthcare (corresponding symbol) this was learned:* *: patient;*  *: relative;* *: healthcare professional;* *: healthcare community.*
